# Atypical sideways recognition of CD1a by autoreactive γδ T cell receptors

**DOI:** 10.1038/s41467-022-31443-9

**Published:** 2022-07-05

**Authors:** Marcin Wegrecki, Tonatiuh A. Ocampo, Sachith D. Gunasinghe, Anouk von Borstel, Shin Yi Tin, Josephine F. Reijneveld, Thinh-Phat Cao, Benjamin S. Gully, Jérôme Le Nours, D. Branch Moody, Ildiko Van Rhijn, Jamie Rossjohn

**Affiliations:** 1 Infection and Immunity Program and Department of Biochemistry and Molecular Biology, Biomedicine Discovery Institute, Monash University, Clayton, Victoria Australia; 2 Division of Rheumatology, Inflammation, and Immunity, Brigham and Women’s Hospital and Harvard Medical School, Boston, MA US; 3 European Molecular Biology Laboratory (EMBL) Australia Node in Single Molecule Science, School of Medical Sciences, University of New South Wales, Sydney, New South Wales Australia; 4 Department of Infectious Diseases and Immunology, Faculty of Veterinary Medicine, Utrecht University, Utrecht, Netherlands; 5 Institute of Infection and Immunity, Cardiff University, School of Medicine, Heath Park, Cardiff, UK

**Keywords:** Innate immunity, X-ray crystallography, T-cell receptor, Antigen processing and presentation

## Abstract

CD1a is a monomorphic antigen-presenting molecule on dendritic cells that presents lipids to αβ T cells. Whether CD1a represents a ligand for other immune receptors remains unknown. Here we use CD1a tetramers to show that CD1a is a ligand for Vδ1^+^ γδ T cells. Functional studies suggest that two γδ T cell receptors (TCRs) bound CD1a in a lipid-independent manner. The crystal structures of three Vγ4Vδ1 TCR-CD1a-lipid complexes reveal that the γδ TCR binds at the extreme far side and parallel to the long axis of the β-sheet floor of CD1a’s antigen-binding cleft. Here, the γδ TCR co-recognises the CD1a heavy chain and β2 microglobulin in a manner that is distinct from all other previously observed γδ TCR docking modalities. The ‘sideways’ and lipid antigen independent mode of autoreactive CD1a recognition induces TCR clustering on the cell surface and proximal T cell signalling as measured by CD3ζ phosphorylation. In contrast with the ‘end to end’ binding of αβ TCRs that typically contact carried antigens, autoreactive γδ TCRs support geometrically diverse approaches to CD1a, as well as antigen independent recognition.

## Abstract (web summary)

T cell receptors are generally thought to contact antigens presented in an end to end configuration. Here the authors show a geometrically alternate sideways mode of recognition of the antigen-presenting molecule CD1a by a γδ T cell receptor.

## Introduction

T cells are subdivided into three main lineages based on the genes encoding their T cell antigen receptors (TCRs), namely, αβ, γδ and γμ T cells^1^. αβTCRs recognise antigens (Ags) encompassing peptides, lipids, and metabolites that are presented by Major Histocompatibility Complex (MHC), CD1 and MR1 molecules, respectively^2^. The total αβ and γδ T cell pools in adult humans are both large in size, but studies of T cell-mediated immunity have mostly focused on αβ T cell recognition and activation. αβ TCRs bind to MHC, CD1, and MR1 with a conserved ‘end to end’ docking mode: the long axes of both proteins are aligned so that the membrane distal surface of the TCR contacts the membrane distal surface of the antigen presenting molecule^3–5^. Although individual αβ TCRs show differential rotation or lateral translation on the surface of antigen presenting molecules, the ‘end to end’ approach means that they essentially bind atop of the Ag-binding cleft and in so doing contact exposed peptide antigen^2^.

For γδ TCRs the number and nature of known antigenic targets continue to expand, and any general principles for the geometry of γδ TCR approach or conserved contact points on their targets are yet to be elucidated^6^. For example γδ TCRs can directly bind many cell surface and soluble proteins, including butyrophilins, ephrin type-A receptor 2 (EphA2), annexin A2, as well as MHC-I like molecules, such as endothelial protein C receptor (EPCR), T10, T22, MR1 and CD1^7–11^. It was recently demonstrated that a population of γδ TCRs does not use the end to end mechanism and instead binds to the underside of MR1^8^. Whether γδ TCRs can adopt unusual approaches to antigen-presenting molecules remains unknown.

Here we sought to understand if γδ T cells recognise the human CD1a protein, which is normally expressed at high density on Langerhans cells and myeloid dendritic cells. CD1 proteins bind and present lipids, including phospholipids, glycolipids, lipopeptides, and apolar ‘headless’ lipids that function as antigens for T cells^12,13^. Typically, the hydrophobic moieties of lipids are buried within the Ag-binding cleft of CD1, whereas polar moieties tend to protrude from the cleft and contact TCRs^14^. Understanding the separate functions of the four human CD1 isoforms is important, because CD1a, CD1b, CD1c and CD1d all have differing patterns of expression in tissues, subcellular trafficking^15,16^ and three-dimensional architecture of the antigen-binding clefts^17–20^. For example, while CD1c has an open, solvent-exposed antigen-binding cleft^19^, CD1a possesses a binding cleft that is sequestered by the A’-roof of CD1a, which partially covers and shields the lipid from TCR contact^21^. These differences result in isoform-related preferences in the repertoire of lipids that can be presented, as well as offer differing binding platforms for TCR recognition.

As contrasted with their roles in αβ T cell function, presently, our understanding of the extent to which CD1 family members represent ligands for γδ T cells is limited. CD1a-dependent activation of γδ T cells from lungs was seen in one study^22^. Several tetramer studies showed that CD1b, CD1c, and CD1d represent bonafide γδ T cell ligands^23–25^, and two structural reports showed how Vδ1^+^ γδ TCRs bound over the CD1d-antigen binding cleft while co-contacting the exposed polar headgroup of the lipid antigen in an ‘end to end’ binding mode that is comparable to αβTCR MHC docking^7,26^. However, CD1a interactions with γδ TCRs have not been reported in part because CD1a presented Ags remain poorly understood, and no immunodominant antigens, equivalent to α-galactosyl ceramide for CD1d, were known.

Normally, antigens are needed for tetramer studies to achieve high avidity binding to TCRs^27^. However, two recent studies^28,29^ show that CD1a carrying mixed endogenous lipids (CD1a-endo tetramers) could readily detect human αβ T cells without adding any defined antigens, bypassing a key technical barrier. Here, using the new approach of CD1a-endo tetramers, we discover autoreactive Vδ1^+^ γδ T cells that are restricted to CD1a. We show that γδ TCRs bind CD1a regardless of nature of the lipid bound, and that the unprecedented approach of Vδ1^+^ γδ TCR recognition, which contacts the backside of the CD1a binding cleft and β2 microglobulin, points to diverse modes of γδ TCR approach to targets.

## Results

### Discovery of CD1a-specific γδ T cells

**Fig. 1:**
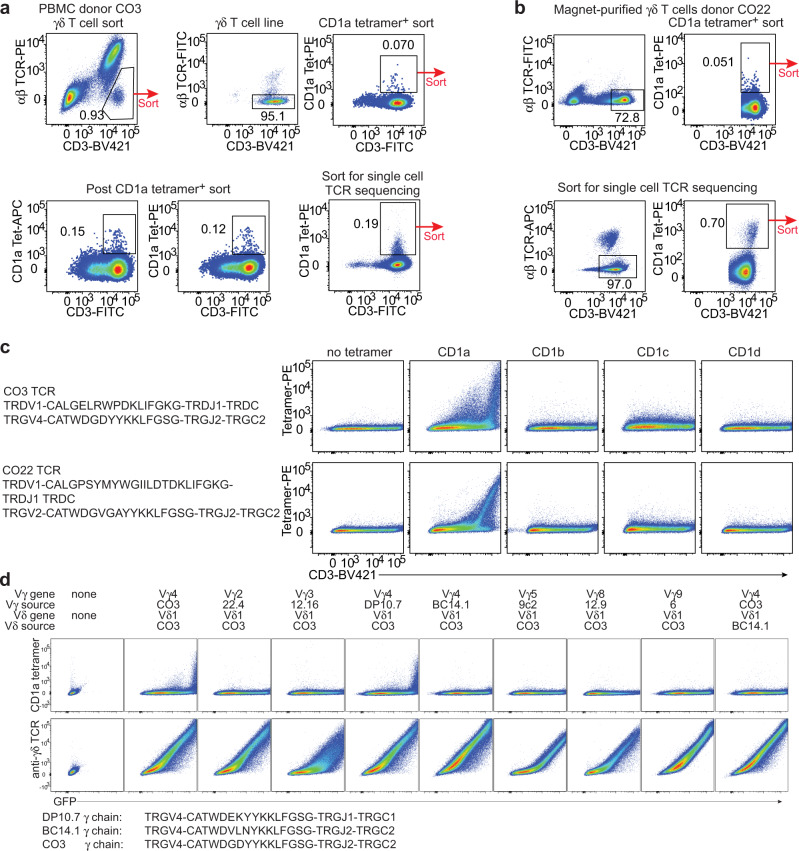
Discovery of CD1a-specific γδ T cells and TCR validation. The gating and sorting strategy that preceded the single cell sort that led to the identification of the CO3 TCR from healthy donor CO3 (**a**) or the CO22 TCR from healthy donor CO22 (**b**) is shown. The CO3 or CO22 TCR was transfected into 293 T cells, together with the CD3 complex, and stained with CD1a, CD1b, CD1c, or CD1d tetramers and an antibody against CD3 (**c**). Results in **c** are representative of 2 experiments. **d** The unmodified CO3 TCR was transfected, or its Vγ4 γ chain was replaced with the indicated Vγ chains from CD1b or CD1d-specific γδ TCRs, or its Vδ1 chain was replaced with the Vδ1 chain of the CD1b-specific BC14.1 TCR. The indicated tyrosine residue at position 104 of γTCR chain was present in CO3 and DP10.7. The transfected cells were stained with CD1a tetramer (top row) or an antibody against the γδ TCR.

While MHC tetramers require loading with a specific peptide to stain αβ T cells, CD1a-endo tetramers can permit binding to autoreactive αβ T cells without knowledge of the carried lipid^29^. This approach allowed discovery of T cells that bind and recognise the membrane-distal roof domain of CD1a itself^29–31^. We reasoned that this new tool might isolate autoreactive CD1a-restricted γδ T cells. To determine if CD1a-binding γδ T cells exist in humans, we first generated a γδ T cell line from peripheral blood mononuclear cells (PBMCs) from a healthy donor, CO3, by flow cytometric sorting of TCR αβ^–^ T cells and CD1a-endo tetramer^+^ T cells (Fig. 1a). In a different donor, CO22, γδ T cells were first enriched by magnetic sorting, followed by FACS sorting of CD1a-endo tetramer^+^ cells (Fig. 1b). In both cases, cells did not expand extensively in vitro, which is a known feature of human γδ T cells^32^ but the staining pattern clearly suggested the presence of CD1a-specific γδ T cells, and we could recover one TCRγ and one TCRδ sequence from each sort at the single cell level (Fig. 1c).

Although Vδ1^+^ γδ T cells are typically less abundant in blood than Vδ2^+^ γδ T cells, both CO3 and CO22 γδ TCRs used Vδ1 and varied in their Vγ chain usage. Transient transfection of the CO3 (Vγ4 Vδ1) and CO22 (Vγ2 Vδ1) γδ TCRs into HEK293T cells demonstrated that both γδ TCRs bound to CD1a-endo tetramer, but not to CD1b-endo, CD1c-endo, or CD1d-endo tetramers (Fig. 1c). CD1a is nearly non-polymorphic in humans, but the differential staining between the CO3 and CO22 γδ TCRs towards CD1a-endo suggested that varied Vγ gene and CDR3 sequence could impact on CD1a-endo reactivity. Overall, these data demonstrate that autoreactive CD1a-specific γδ T cells exist, albeit at low frequency in the peripheral blood of healthy human subjects.

### TCR γ and δ chains co-recognise CD1a

There are several reports of Vδ-chain dominance, particularly Vδ1, in the mechanism of target recognition by γδ T cells^7,26,33,34^^,^. To test whether the CO3 γδ TCR also depends on the Vδ chain for CD1a recognition, we replaced its Vγ4 chain with Vγ2, Vγ3, Vγ5, or Vγ9 chains from other γδ TCRs. We also used distinct Vγ4 chains from the CD1d-specific γδ TCR DP10.7 and the CD1b-specific γδ TCR BC14.1 and transiently transfected all hybrid γδ TCRs into HEK293T cells (Fig. 1d). Even though all the hybrid γδ TCRs were expressed at similarly high levels, only the native CO3 Vγ chain and the DP10.7 Vγ chain could support CD1a staining in combination with the CO3 Vδ chain. Of note, the BC14.1 Vγ chain, which did not support CD1a-endo staining, differs only in 3 amino acids from the CO3 Vγ chain, while the DP10.7 Vγ chain differs by only 2 amino acids, suggesting that tyrosine at position 104 (Tyr104) within the CDR3γ loop is essential for CD1a-endo recognition. Replacement of the CO3 Vδ1 chain with the BC14.1 Vδ1 chain, did not lead to CD1a-endo tetramer staining, suggesting that the CDR3δ loop of the CO3 γδ TCR contributes to CD1a-endo binding. Accordingly, both γ and δ CO3 TCR chains are required for CD1a-endo binding and Tyr104 is apparently essential. Overall, evidence suggests that Vδ1 are most frequently observed for binding CD1b^23^, CD1c^25^, and CD1d^7,26^ and now CD1a (Fig. 1).

### γδ TCR binding to CD1a does not require specific lipid ligands

**Fig. 2:**
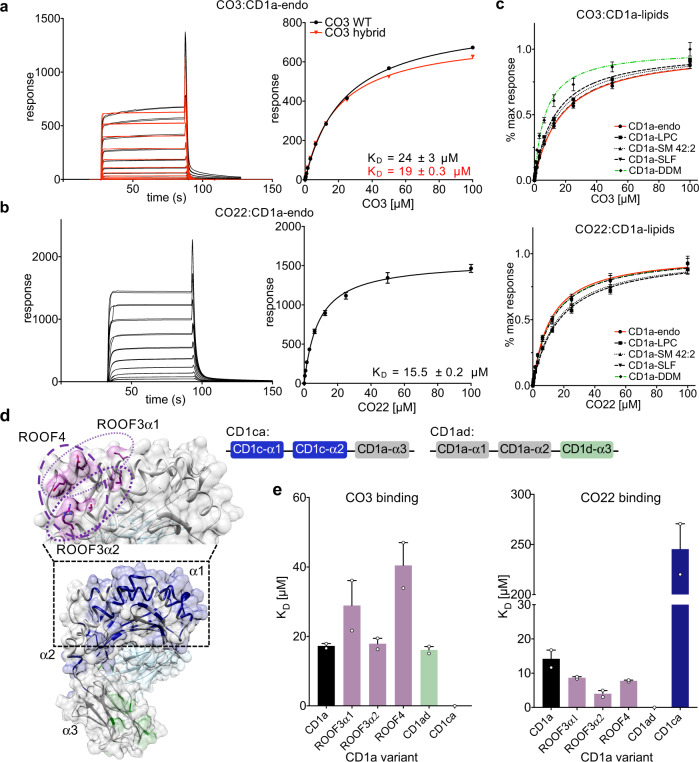
CD1a binds γδ TCRs in vitro. Surface plasmon resonance (SPR) sensorgrams and calculated binding curves for CO3 (**a**) and CO22 (**b**) γδTCRs injected over a flow cell containing CD1a-endo. Comparison between HEK293S-cells produced (black) and refolded hybrid (red) TCR is shown for CO3 γδTCR. **c** SPR data showing normalised binding curves between CD1a-endo (red curve) or loaded with different ligands (self-lipids: black symbols, DDM: green) to CO3 (top) and CO22 (bottom) γδTCRs. The curves shown in **a**–**c** are representative of one experiment. Dissociation constants were calculated from two independent experiments (*n* = 2). For each concentration the points represent the mean and the error bars correspond to SD. **d** Engineered CD1a variants used to elucidate the binding determinants of the γδ TCRs. The side chains of mutated residues across the A’ roof (top) are shown in pink. The domain-swap chimeras (bottom) contain residues corresponding to human CD1c (CD1ca: navy blue) and human CD1d (CD1ad, light green). Light grey areas correspond to wild-type CD1a residues. The origin of the α domain in each construct is shown in the box diagram. **e** Dissociation constants of the binding between CO3 (left) or CO22 (right) γδ TCRs and wild-type CD1a (black), CD1a A’ roof mutants (pink), CD1ad (green), and CD1ca (navy blue) chimeric proteins were calculated from two independent experiments. The error bars correspond to SEM. Source data are provided as a Source Data file.

To understand the mechanism underpinning recognition of human CD1a by Vδ1^+^ γδ T cells we first expressed recombinant CO3 and CO22 γδ TCRs using a mammalian expression system, purified these γδ TCRs to homogeneity, and measured the affinity of the interaction towards CD1a-endo using surface plasmon resonance (SPR). The CO3 and CO22 γδ TCRs bound CD1a-endo with dissociation constant (K_D_) of 23.6 ± 3.1 μM and 15.5 ± 0.5 μM, respectively (Fig. 2a, b). To our knowledge TCR cross-reactivity across other CD1 isoforms is undescribed. Nearly all previously identified TCRs are directed at the α1–α2 domains that show relatively low (<40%) amino acid sequence identity^2^. However, α3 domains of CD1 proteins showed higher sequence similarity, and the CD1a-reactive CO22 γδ TCR showed weak but clearly detectable binding (K_D_ > 100 μM) to human CD1c (Supplementary Fig. S1a).

The cross-reactivity with CD1c provided the first hint of a non-canonical binding mode, which was tested further with sphingomyelin (SM) and sulfatide (SLF). Recent studies have shown that the headgroup of a very long chain C42 diene sphingomyelin (42:2 SM) protrudes from the membrane distal end of CD1a and broadly blocks polyclonal αβ TCRs in all donors tested^29^. Sulfatide also acts at a known site on CD1a, where it blocks T cell response through local remodelling of a triad of residues on the membrane distal end of CD1a near the F’ portal. To determine the role of lipids in promoting and blocking the γδ TCR-CD1a interaction, we undertook affinity measurements using CD1a loaded with two known antigens, lyso-phosphatidylcholine (LPC) and di-deoxymycobactin lipopeptide (DDM)^35^, as well as two known TCR blockers, sulfatide (SLF) and 42:2 SM (Fig. 2c)^29^. DDM is a CD1a-restricted lipopeptide derived from *Mycobacterium tuberculosis*, which protrudes from the F’ portal located on one side of CD1a and activates certain bacteria-specific T cell clones and can block activation of other autoreactive αβ T cell clones^31,35,36^. In contrast to strong effects seen previously for αβ TCRs, 42:2 SM and SLF did not inhibit γδ TCR binding to CD1a (K_D_ range 15–22 μM and 10–13 μM for CO3 and CO22 γδ TCRs, respectively), and the antigens LPC and DDM did not substantially augment binding to CD1a (Fig. 2c and Supplementary Table 1). Of interest, the binding of both γδ TCRs to CD1a-DDM showed a modest increase in affinity. For example, the CO3 γδ TCR binding towards CD1a-DDM was 9 μM versus 24 μM for CD1a-endo. Collectively, this suggested that autoreactive γδ TCRs are permissive to a wide range of lipid ligands for CD1a, including a SM ligand that broadly blocks αβ TCR binding.

### Mutational analysis of CD1a identifies differing effects on αβ and γδ TCRs

The lack of a significant lipid-mediated blocking or augmenting effect on γδ TCR-CD1a binding suggested that the two CD1a-autoreactive γδ TCRs might follow the recently described pattern of a ‘lipid-agnostic’ CD1a recognition by autoreactive αβ T cells^28,30^. Here, many CD1a-restricted αβ T cells are thought to target the membrane distal end of CD1a, and that ‘end to end’ interactions of CD1a and TCR are abolished in the context of single and multiple mutations in the α1 and α2 helices of the heavy chain of CD1a, which constitute the membrane distal face of CD1a^28^ (Fig. 2d). To test γδ TCR recognition of the distal end of CD1a, we used a panel of CD1a A’-roof mutants and measured the affinity of interaction between CD1a-endo and the two γδ TCRs. We previously found that triple mutants of the CD1a roof block binding of αβ TCRs without globally changing roof structures in ways that affect lipid binding^28^. Here we found that triple mutants across the A’-roof (ROOF3α1: E62A, E65A, I72A; ROOF3α2: I157A, T165A, R168A; and ROOF4: E62A, E65A, T165A, R168A) were still recognised well by both γδ TCRs with affinities of 40 μM or higher (Fig. 2e and Supplementary Fig. S1b). Overall, our SPR-based analyses pointed towards a recognition mechanism by the autoreactive γδ TCRs that was largely independent both of lipids and the adjacent membrane distal surface of human CD1a, although moderate increase in affinity of autoreactive TCRs to DDM was observed.

### The two γδ TCRs have a differing dependence on the CD1a α3-domain

Nearly all αβ and γδ TCRs bind ‘end to end’ with antigen presenting molecules, so that the TCR resides atop the antigen-binding cleft and contacts carried antigen, but γδ TCR recognition of CD1a has not been previously studied. Thus, the lipid and α1-α2 domain independence could be explained if γδ TCRs bind CD1a, but do not bind on its membrane distal roof surface. The possibility of an alternative docking mode was supported by one recent study in which a subset of human γδ TCRs can directly recognise the α3 domain of MR1^8^. To test whether the α3 domain of CD1a was recognised, we engineered chimeric CD1 proteins: CD1ad, carrying the α1 and α2 domains of CD1a and the α3 domain from human CD1d, as well as a CD1ca protein, with CD1c-derived α1 and α2 domains fused to the α3 domain of CD1a (Fig. 2d). SPR showed that the CO3 γδ TCR could recognise CD1ad (K_D_: 16.1 ± 1 μM) but not the CD1ca protein, indicating that CD1a α1-α2 domains were essential and the CD1a α3 domain was dispensable for the CO3 γδ TCR interaction (Fig. 2e). However, the binding of the CO22 γδ TCR was impaired in the case of CD1ad and still detectable when recognising CD1ca (K_D_ > 100 μM) (Fig. 2e and Supplementary Fig. S1c). These data suggested that the CO22 γδ TCR and CO3 γδ TCR interacted with CD1a via different mechanisms, with the CO22 γδ TCR being dependent on the α3-domain, whereas the CO3 γδ TCR was not.

### An unprecedented mode of γδ TCR recognition

To understand the molecular basis underpinning γδ TCR recognition of CD1a, we determined the crystal structure of the CO3 γδ TCR alone at 2.0 Å and in complex with CD1a-endo, CD1a-sulfatide and CD1a-DDM to a resolution of 3.2 Å, 2.7 Å and 3.0 Å, respectively (Supplementary Table 2). Here, to crystallise these ternary structures, we engineered a hybrid TCR fusing the variable CO3γ domain to the constant TCR β domain and the variable CO3δ domain to the constant TCR α domain, as done previously^8,37^. As shown by the SPR data, the resulting hybrid TCR bound human CD1a-endo with an affinity value (K_D_ = 19 ± 0.3 μM) comparable to ‘wild-type’ CO3 γδ TCR (K_D_ = 23.6 ± 3.1 μM) (Fig. 2a). The crystal structure at the CO3 γδ TCR-CD1a interface was unambiguous, permitting a detailed analysis of the intermolecular contacts. The comparison between CD1a-bound and CO3 γδ TCR binary structures indicated minor conformational changes within the Vγ4 domain of the CO3 γδ TCR. The most notable conformational change corresponded to the hypervariable 4 (HV4) region, which is thought to bind butyrophilin-like 3 protein and remained solvent exposed in the CD1a-CO3 γδ TCR complex (Supplementary Fig. S2)^38^. The three ternary structures were very similar, so the structural analyses, unless explicitly stated, focus on the highest resolution structure, CO3 γδ TCR-CD1a-sulfatide.

**Fig. 3:**
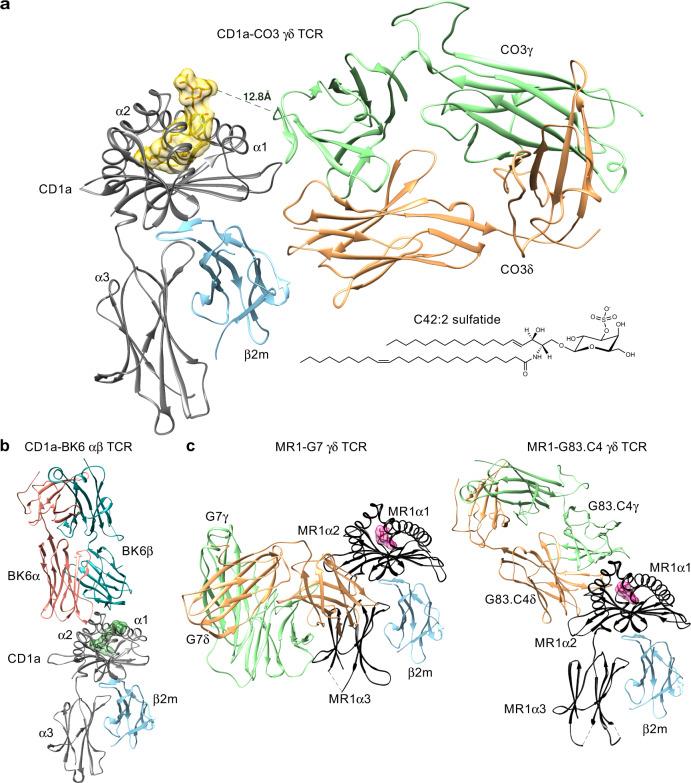
CO3 γδ TCR binds CD1a in an unprecedent manner. **a** The overview of the crystal structure of CO3 γδ TCR-CD1a-sulfatide is shown. Both chains of the CO3 γδ TCR (γ chain in green; δ chain in orange) docked on the side of CD1a (grey) - β2m (blue) heterodimer forming a ~90° incident angle. The lipid antigen sulfatide (yellow) protrudes from the cleft of CD1a. The sideways docking mode is contrasted with **b** CD1a-restricted autoreactive αβ TCR BK6 (α chain in coral, β chain in cyan, lipid ligand in dark green; PDB: 4X6C) and **c** the docking modes seen for MR1 restricted γδ TCRs: G7 (left: PDB 6MWR) and G83.C4 (right: 7LLI). MR1 is shown in black, γ chains are in green, δ chains in orange and the MR1 ligands in pink.

The CO3 γδ TCR-CD1a-sulfatide complex revealed an unexpected mode of γδ TCR docking onto an antigen-presenting molecule (Fig. 3a). Namely, the CO3 γδ TCR bound CD1a-β2 m heterodimer with an ‘end to side’ docking mode whereby the 87.4° incident angle is nearly perpendicular with CD1a. The TCR bound on the α1 side of the F’-pocket, where it also contacted β2-microglobulin but not the lipid ligand (Fig. 3a). This sideways docking mode differed substantially from the typical mechanism of αβTCRs binding to MHC and MHC-I like molecules, as well as the two available CD1d- γδ TCR structures^7,26^, where ‘end to end’ docking occurs with the main axis of the TCRs showing an incident angle of 7–10° with the target protein and direct contact with the carried antigen (Fig. 3b)^39^. Moreover, the CO3 γδ TCR-CD1a docking mode was completely distinct from any γδ TCR complex structure previously reported, including the recently described Vδ1^+^ and Vδ3^+^ TCRs in complex with human MR1^8,37^ (Fig. 3c). For MR1 the Vδ1^+^ γδ TCR sat underneath the antigen-binding cleft and contacted the α3-domain, whereas the Vδ3^+^ γδ TCR obliquely bound to the MR1 antigen-binding cleft. For CD1a, the striking features of the CO3 γδ TCR mode of contact are binding below the antigen display platform and the high incidence angle, which define a ‘sideways’ approach, as well as substantial contact with β2-microglobulin, rather than the α3 domain.

### γδ TCR co-recognises CD1a heavy chain and β2m

**Fig. 4:**
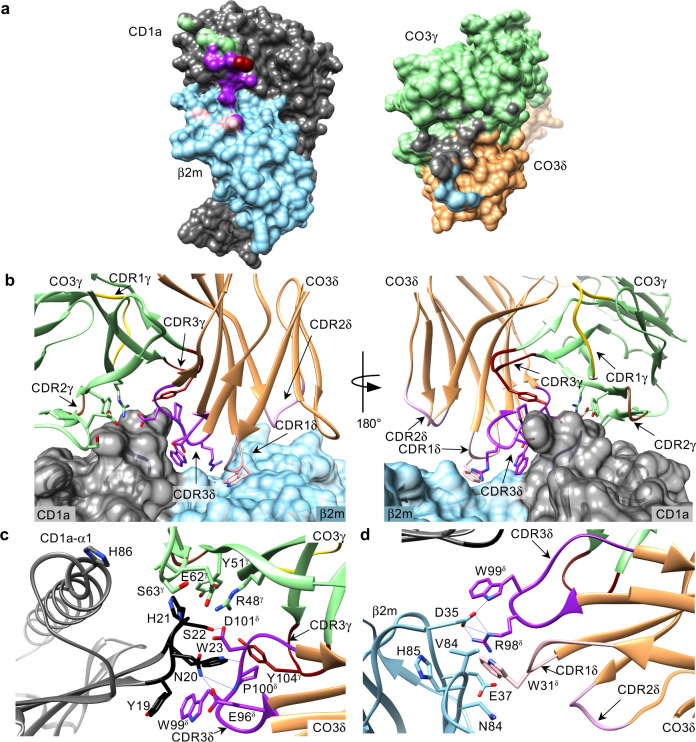
Interactions between CD1a and CO3 γδ TCR. **a** The surface of CD1a (dark grey) and β2m (blue) heterodimer (left) involved in the interaction with CO3 γδ TCR. The area buried by the interaction with CO3 framework (FR) γ is shown in green, with CDR3γ in dark red, with CDR1δ in light pink and with CDR3δ in purple. Within the footprint of the Ag-presenting molecule on the TCR (right, CO3γ in green, CO3δ in orange) 70% of the BSA corresponds to CD1a (grey) and 30% to β2m residues (blue). **b** Both chains of the γδ TCR are involved in the recognition of CD1a/β2m (grey and blue). CO3γ chain contribution is limited to the FR γ region (green) and CDR3γ loop (dark red) and does not involve CDR1γ (yellow) or CDR2γ (brown) loops. CDR3δ loop (purple) from the δ chain (orange) of CO3 penetrates the cavity between the heavy chain and β2m and contributes to 40% of total BSA, CDR1δ (light pink) constitutes additional 20% of the interface area and CDR2δ loop (dark pink) does not participate in the interaction. The side chains of the CO3 residues contacting CD1a/β2m are shown. **c** The side chains of CD1a and the CO3 γδ TCR residues involved in the complex formation are shown and labelled. The core of the interaction corresponds to the 19-23 loop (black) of CD1a (grey) that make extensive hydrogen bonds (blue lines) with the framework region of CO3γ (green), CDR3γ loop (dark red) and CDR3δ loop (purple). **d** The side chains of β2m and the CO3 γδ TCR residues involved in the TCR complex formation are labelled, showing electrostatic interactions between β2m (blue), CDR1δ (light pink) and CDR3δ (purple) loops.

While the Vγ4 domain of CO3 γδ TCR localised atop of the Ag-binding cleft, near the C-terminal end of the α1 helix of CD1a, the variable loops of the Vδ1 domain protruded into the cavity between CD1a-α1 and β2m creating an interface with the shape complementarity score of 0.74, indicating a very good fit (Fig. 4a). The total buried surface area (BSA) was 1320 Å (680 Å^2^ for CD1a and 640 Å^2^ for the TCR) (Fig. 4a), with CD1a contributing 70% and β2 m 30% to the total interface area. In agreement with chain swap experiments (Fig. 1d), both chains of the γδ TCR were involved in ternary complex formation with 40 and 60% contribution of the γ and δ chains, respectively (Fig. 4a). Here, the CDR1δ and CDR3δ loops contributed 20 and 40% BSA to the interface, respectively while the CDR3γ loop contributed 6% (Fig. 4b). The additional 35% of the BSA was attributable to the framework region of the Vγ4 chain, which also agrees with chain swap data showing an essential role of Vγ4 in binding the CO3 γδ TCR (Fig. 1d), while the remaining variable loops (CDR2δ, CDR1γ, and CDR2γ) were not involved in the interaction with CD1a (Fig. 4b).

A central focal point of the CO3 γδ TCR-CD1a-sulfatide complex is dominated by a short loop (residues 19–23) in the α1 domain of CD1a that makes extensive contacts (Supplementary Table 3) with the CO3 γδ TCR, whereby residues from the CDR3γ loop, framework residues from Vγ chain and the CDR3δ loop converged onto this CD1a recognition determinant (Fig. 4c). This short CD1a loop contains three aromatic residues that play a principal role in interacting with the CO3 γδ TCR. Namely, there were aromatic *pi*-stacking interactions between Tyr19 in CD1a and Trp99δ from the CDR3δ loop, with this aromatic cluster being extended by interactions between Trp23 from CD1a and Tyr104γ from the CDR3γ loop (Fig. 4c). The importance of this Trp23-Tyr104γ interaction seen in the three crystal structures was independently identified by the chain swap experiments, in which the only hybrid TCRs with tyrosine at this position were able to stain with CD1a tetramers (Fig. 1d). Moreover, His21 from CD1a made a series of van der Waals interactions with framework (FR) γ residues, Arg48γ, Tyr51γ and Glu62γ and Asp101δ from the CDR3δ loop (Fig. 4c). These germline-encoded framework residues are conserved across all the human TRGV genes except TRGV9, (Supplementary Fig. S3), potentially explaining why the TRGV9 TCRγ chain did not support CD1a binding (Fig. 1d). The contacts between CD1a and the CDR3δ loop were extended by a series of polar interactions, including hydrogen bonds of Glu96δ and Asp101δ with Asn20 and Ser22 from CD1a, respectively, as well as main chain hydrogen bonding of residues 99–100 in the CDR3δ loop with Asn20 and Trp23 from CD1a, respectively (Fig. 4c).

Contacts with β2m were solely mediated by the Vδ chain, using the CDR1δ and CDR3δ loops (Fig. 4d). The contribution of the germline encoded Vδ1 residues involved Trp31δ from CDR1δ loop, which made extensive contacts with Asp35, Glu37 and Asn84 in β2m and corresponded to ~20% of BSA. These interactions were extended by the neighbouring CDR3δ loop, whereupon Arg98δ formed a salt bridge with Asp35, while Trp99δ formed a hydrogen bond to Asp35 from β2m (Fig. 4d). Accordingly, a series of germline-encoded and non-germline encoded regions of the CO3 γδ TCR underpinned this atypical sideways recognition mode with CD1a, which involved γδ TCR co-recognition of CD1a and β2m.

### Antigen-independent binding of CD1a to a γδ TCR

To generate the CO3 γδ TCR-CD1a crystal structure, we used CD1a protein carrying heterogenous human embryonic kidney (HEK) cell-derived endogenous lipids (CD1a-endo) and were not able to unambiguously model lipid antigen in the CD1a cleft. However, the structure of the complex between the CO3 γδ TCR and CD1a-sulfatide, an endogenous lipid known to bind human CD1a^21^, showed a clear lipid density in the cleft of CD1a (Supplementary Fig. S4). The arrangement of the lipid chains highly resembled those observed in the CD1a-42:2 SM structure published recently^29^, where both lipid tails ran in parallel inside the A’-pocket (Supplementary Fig. S4b). The sulfatide headgroup protruded through the F’-portal and adopted a fixed position near the α2-helix of CD1a (Fig. 3a). Whereas sulfatide was previously shown to alter the distal surface of CD1a and protrude to block activation of αβ T cells by CD1a^31^, the location of the blocking headgroup for αβ T cells was 13 Å distant from the atypical CO3 γδ TCR binding site (Fig. 3a). The separate positioning of what are normally blocking headgroups on the ‘top’ of CD1a, versus the TCR contact site on the ‘side’ of CD1a, can explain why neither sulfatide nor 42:2 SM blocked CO3 γδ TCR binding to CD1a (Fig. 2c).

**Fig. 5:**
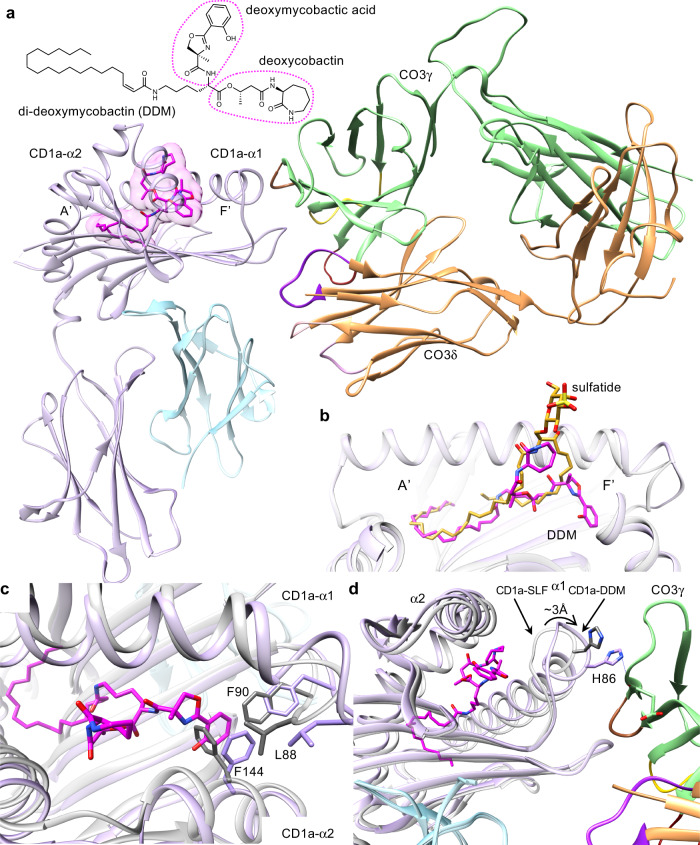
Interaction between CO3 γδ TCR and CD1a carrying diverse lipid antigens. **a** Overview of CD1a-DDM-CO3 ternary structure shows the ligand orientation in the cleft of CD1a (lilac). DDM (magenta) fully occupies A’ and F’ pockets and does not significantly protrude through the F’ portal of CD1a. **b** Relative position of the ligands in the cleft of CD1a bound to sulfatide (yellow) and DDM (magenta) in CD1a-CO3 ternary structures is shown. **c** Comparison of CD1a backbone in CO3-CD1a-sulfatide complex (light grey) and CO3-CD1a-DDM complex (lilac) shows that deoxymycobactic acid moiety of DDM (magenta) expands the F’ pocket of CD1a by conformational changes of hydrophobic residues within the cleft of CD1a. **d** DDM-induced F’ pocket expansion is associated with a 3 Å displacement of the CD1a-α1 helix towards the FRγ region (green) of CO3 leading to a formation of an additional hydrogen bond (blue line) between CD1a^His86^ and the main chain of CO3γ.

Given the large distance between the TCR docking site and the F’ portal, which is the site of antigen protrusion, we sought to understand why another well-known CD1a-restricted antigen, the *M. tuberculosis*-derived lipopeptide DDM, could increase the interaction between CO3 γδ TCR and CD1a (Fig. 2c). Accordingly, we solved the structure of the CO3 γδ TCR bound to CD1a carrying a new synthetic form of DDM that precisely matches the structure of natural DDM^40^ (Fig. 5a), which showed a clear DDM ligand density in the CD1a cleft (Supplementary Fig. S4c). Similar to a prior structure of CD1a bound to a synthetic DDM-like molecule^36^, the C20:1 acyl tail of DDM anchored in the A’ pocket. However, in contrast to CO3γδ TCR-CD1a-sufatide, the DDM lipopeptide head was enclosed almost entirely within the cleft, with the deoxymycobactic acid occupying the F’-pocket and the deoxycobactin moiety in the F’-portal (Fig. 5b). Although the DDM head group was distant from the TCR, its binding to CD1a induced notable rearrangement in the hydrophobic interior (Fig. 5c), where Phe144 in the α2-helix was pushed by the aryl ring of DDM towards Leu88 and Phe90, expanding the cleft and deforming the α1 domain of CD1a that shifted 3 Å towards the Vγ chain of CO3 (Fig. 5d). Consequently, the buried surface area between CD1a and the γ chain of CO3 increased from 290Å^2^ (CD1a-sulfatide) to 390Å^2^ (CD1a-DDM), and His86 in CD1a made additional hydrogen bond with the main chain of CO3γ (Fig. 5d). Accordingly, the DDM antigen can cause an induced fit change within the CD1a cleft, which translated into a change in the outer surface of CD1a that might explain the increase in binding affinity upon complex formation (Fig. 2c).

### Energetic basis underpinning the CO3 γδ TCR-CD1a interaction

**Fig. 6:**
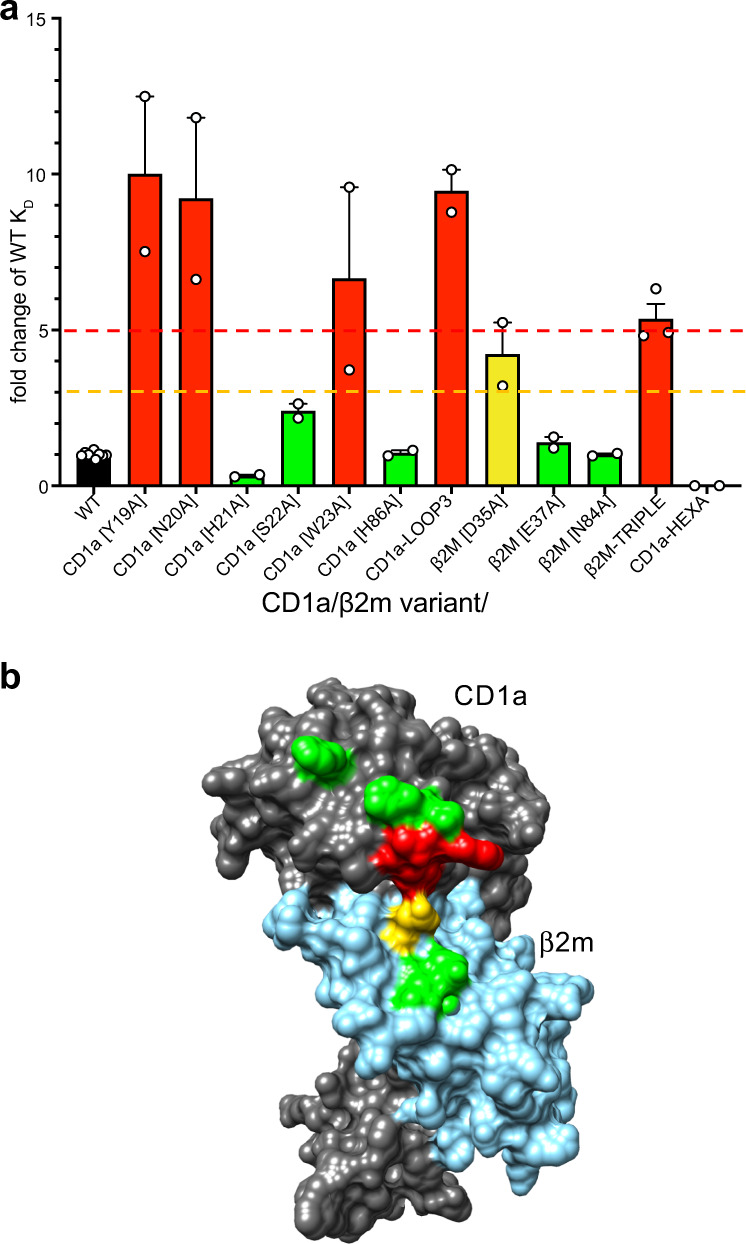
Energetic landscape of CD1a-CO3 γδ TCR interaction. **a** SPR was used to measure the binding affinity between CO3 γδ TCR and CD1a containing single or multiple mutations across the CO3-CD1a complex interface. Green (K_D_ change < 3-fold), orange (3 to 5-fold decrease in K_D_) and red (>5-fold decrease in K_D_) bars summarise the change in the interaction between CO3 γδ TCR and CD1a mutants with respect to the wild-type CD1a protein (black bar, K_D_ = 24 μM). The values were calculated from at least two independent experiments (*n* ≥ 2). The error bars correspond to SEM. **b** Residues involved in the CO3-CD1a complex formation on the surface of CD1a (dark grey)/β2m (blue) are coloured based on the mutation analysis shown in **a**. Amino acids critical for the recognition (red) by CO3 γδ TCR correspond to the Tyr19-Trp23 loop of CD1a. Source data are provided as a Source Data file.

Given the unusual nature of the CO3 γδ TCR-CD1a complexation, we next aimed to investigate the energetic basis underpinning this interaction. We generated additional alanine-scanning mutations on the CD1a-β2 m heterodimer, which tested key candidate interactions identified by the crystal structure. These changes included six single site alanine mutations on the CD1a heavy chain (Tyr19, Asn20, His21, Ser22, Trp23, and His86) and three on β2m (Asp35, Glu37, Asn84). Then we assessed the impact of binding to the CO3 γδ TCR by SPR (Supplementary Fig. S5). The mutants were categorised as having no effect (green) if the K_D_ change was within 1 to 3-fold the WT value (24 μM), moderate effect (yellow) for the K_D_ change 3 to 5-fold, or strong effect (red) if the K_D_ value showed a > 5-fold change (Fig. 6a). We found markedly decreased binding to CD1a mutants changed at the positions Tyr19, Asn20, Trp23 of CD1a and Asp35 of β2 m (Supplementary Fig. S5). However, none of the point mutations completely disrupted binding. Therefore, we generated two triple mutants located near the proposed TCR contact sites, namely CD1a-LOOP3 (Tyr19, His21, Trp23) and β2m-TRIPLE (Asp35, Glu37, Asn84), both of which further reduced CO3 γδ TCR binding (Supplementary Fig. S5). Finally, a CD1a-β2m heterodimer carrying mutations at all 6 positions (CD1a-HEXA) did not detectably bind to CO3 γδ TCR (Supplementary Fig. S5). Overall, the binding data confirm that the interaction sites seen in crystal structures control the binding of CO3 γδ TCR to CD1a, whilst defining the particular residues within the CD1a 19-23 loop and the β2m chain that represent key energetic hot spots underpinning this interaction (Fig. 6b).

### γδ TCR-CD1a binding induces TCR clustering and CD3ζ phosphorylation

We next investigated whether the atypical direction of TCR engagement led to T cell signal transduction. First, we stably expressed CO3 γδ TCR, CO22 γδ TCR and a positive control CD1a-autoreactive αβTCR called BK6 in Jurkat76 cell lines^30^. As expected, co-culture with K562 cells expressing CD1a increased the frequency of Jurkat76.BK6 CD69^+^ cells. For the γδ TCRs, we noted a high background CD69 expression, and no clear increase after TCR transfer and CD1a^+^ cell addition (Supplementary Fig. S6a). This outcome was expected, in so far as human γδ Τ cells^32^, including MR1-reactive TCRs with unusual docking angles^8^, expand or signal poorly ex vivo. In mice, hypo-responsive γδ TCR signalling pathways have been broadly observed in vivo^41^, where hypo-responsiveness protects against negative selection. However, for mouse γδ T cells proximal signalling via TCRζ is preserved in ways that allow maintenance of cells and expansion in immunosurveillance niches^42^. The alternative possibility is that the high incidence binding angles and ‘head to side’ interactions, might not lead to productive TCR clustering and signalling.

**Fig. 7:**
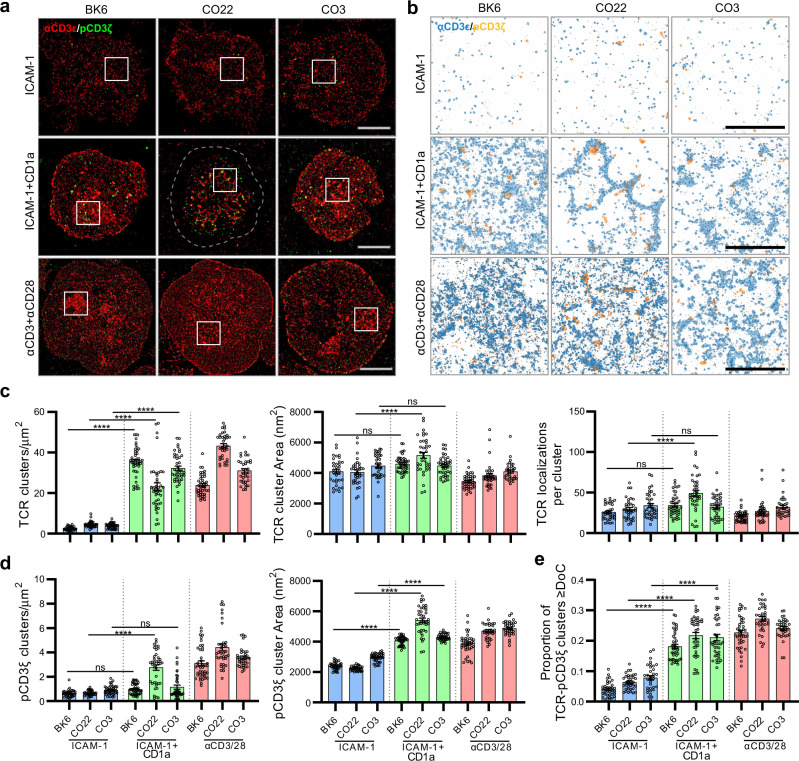
CD1a-induced γδ TCR clustering and signalling. **a** Dual-colour single-molecule localisation microscopy images of CD3ε (red) and pCD3ζ (green) in Jurkat T cells expressing CD1a-reactive BK6, CO22 and CO3 TCRs triggered on supported lipid bilayer decorated with ICAM-1, ICAM-1 + CD1a or anti-CD3/CD28 antibodies under 5 min. Scale bar 5 µm. **b** Closeup view (4 × 4 µm) of cluster maps generated through DBSCAN analysis based on representative regions (boxed) from the corresponding single-molecule localisation microscopy images. Pseudo coloured CD3ε and pCD3ζ clusters are shown in blue and orange respectively. Localisations outside the threshold of clustered organisation are indicated in black dots. Scale bar 2 µm. **c**, **d** CD3ε and pCD3ζ clustering was quantified by DBSCAN analysis and the degree of CD3ε-pCD3ζ colocalisation analysed with DoC thresholding and reported as proportion of colocalised clusters **e**. Analysis was performed across *n* ≥ 30 single T cells which includes at least three independent replicates. Statistical significance determined by one-way ANOVA; ns, not significant; *P ≤ 0.05; **P ≤ 0.01; ***P ≤ 0.001; ****P ≤ 0.0001. The exact values of the P values are shown in Supplementary Table 4. Error bars represent the SEM. Source data are provided as a Source Data file.

Therefore, we investigated TCR clustering using antibodies against CD3ε subunits and CD3ζ phosphorylation upon exposure to CD1a. We exposed Jurkat cell lines to supported lipid bilayers containing either ICAM-1 only (negative control) or ICAM-1 and CD1a or anti-CD3/anti-CD28 antibodies that we used as a positive control. We analysed single-molecule images (Fig. 7a) and DBSCAN cluster maps (Fig. 7b, c). Unlike the pattern of CD69 expression, we observed significant increase in the density of TCR clusters for both αβ and γδ TCRs at rates similar to anti-CD3 antibody positive controls (Fig. 7c). Both unstimulated (ICAM-1 only) and stimulated (ICAM-1 and CD1a or anti-CD3/CD28) TCRs exhibited a non-random distribution on the cell membrane, as indicated by a significantly larger L(*r*)-*r* value relative to complete spatial randomness in Ripley’s K analysis (Supplementary Fig. S6b). Further, L(*r*)-*r* values recorded for CD1a-binding induced clustering were larger than unstimulated TCRs but plateaued at a lower value than anti-CD3/CD28 stimulated TCRs. In addition, the CO22 γδ TCR showed larger TCR clusters after addition of CD1a. Thus, CD1a binding-induced spatial reorganisation of all CD1a-specific TCRs was prevalent irrespective of any geometric constraints imposed by docking topologies.

CD1a-induced phosphorylation of the CD3 signalling complex was examined by co-staining of anti-TCR with anti-pCD3ζ, which binds specifically to phosphorylated ITAM domain epitope Tyr142 in each CD3ζ chain. Cluster density increased significantly for the CO22 TCR after CD1a addition (Fig. 7d), and all three TCRs showed a significant ~two-fold increase in cluster size after adding CD1a or CD3/CD28. Combining the approaches to analyse TCR and pCD3ζ cluster colocalisation, a degree of colocalisation (DoC) score was implemented based an analysis strategy known as combined cluster detection and colocalisation (Clus-DoC). In these experiments the DoC threshold for colocalisation was set to DoC ≥ 0.1, above which the values represent colocalisation events captured between TCR and pCD3ζ. Accordingly, nearly 20% of all TCR clusters showed a DoC score above the colocalisation threshold when triggered by CD1a binding (Fig. 7e). The fraction of TCRs colocalised with pCD3ζ are signalling competent and involved in T cell signal propagation. In previous work^43^, densely packed TCR clusters were more signalling competent and involved in signal propagation. Of CD1a-specific TCRs examined here, CO22 forms the densest TCR clusters which is indicated by the highest number of TCR localisations in clusters (Fig. 7c) and the highest degree of colocalisation with pCD3ζ (Fig. 7e), suggesting higher signalling capacity for CO22 TCR compared to BK6 and CO3. Overall, despite unusual CO3 and CO22 γδ TCR-CD1a docking topologies, their interactions led to initiation of proximal signalling, comparable to that of BK6 TCR-CD1a engagement.

## Discussion

Whereas antibodies directly recognise diverse antigens, αβ TCRs co-recognise antigenic fragments that are co-presented on the membrane distal surface of antigen-presenting molecules. Key aspects of this T cell interaction model, whereby TCRs bind ‘end to end’ with antigen presenting molecules and the small antigens carried, apply also to CD1 and MR1, where αβ TCR-lipid-CD1 and αβ TCR-metabolite-MR1 are key mechanisms of recognition^14^. Nevertheless, exceptions to this TCR co-recognition paradigm are emerging, as autoreactive TCRs restricted to CD1a and CD1c can directly contact the Ag-presenting molecules, while not co-contacting the carried lipid ligand^30,44^. In previously studied examples, CD1c-restricted TCRs sat centrally atop the antigen-binding cleft, and only small, headless ligands allowed TCR binding as they were fully enclosed within CD1c. For CD1a, autoreactive TCRs bind in the ‘end to end’ manner but contact a large A’-roof structure on CD1a that is distinct from the protruding lipid headgroups^30,44^. However, despite these fundamental differences in TCRs contacting or not contacting the carried antigen, the membrane distal end of the TCR typically docks ‘end to end’ on the α-helices at the membrane distal segment of the antigen presenting molecule.

Here we provide the first insights into γδ TCR recognition of CD1a, which along with other recently discovered γδ TCR targets, such as CD1b and MR1, now raise the basic questions about whether the ‘end to end’ mode of recognition used by αβ T cells generally applies to γδ T cells. The question also arises as to whether TCR recognition of proteins that are normally thought of as ‘antigen presenting molecules’ actually represent antigen presentation. Some CD1d-reactive γδ TCRs recognise glycolipids with a ternary ‘end to end’ interaction, leading to the idea that rearranged TCRs recognise normally diverse glycolipids^7,26^. However, other CD1d reactive γδ TCRs do not require glycolipid antigen to be activated by CD1d^7,26^. Furthermore, other γδ TCR targets like the EPCR, as well as butyrophilins, butyrophilin-like molecules, EphA2, annexin A2, and phycoerythrin are not known to present small carried antigens^6,10,11,38,45^. Here we identify a γδ TCR target, CD1a, that is normally considered an antigen presenting molecule, yet does not function to display carried lipid antigen to the TCRs identified.

Four striking features of the mechanism identified here are the high TCR angle of incidence; the TCR contact site located below the antigen display platform; co-recognition of β2m; and low effects of lipid antigens and blockers on γδ TCR binding to CD1a. Thus, recognition of CD1a by the CO3 γδ TCR can be considered a sideways mechanism that functions outside the paradigmatic ‘end to end’ mode of recognition, and this mechanism does not represent lipid presentation. This conclusion is likely also true for CO22 γδ TCR, which although its ternary structure was not determined, binds CD1a in a lipid-independent, α3-domain dependent mechanism. We do acknowledge that the frequency of this unusual mechanism among γδ T cells is not yet known. However, very recent studies show that γδ TCR recognition of CD1b likewise does not require exogenous antigen^34^. Also, recently identified MR1-reactive γδ TCRs do not require any carried antigens^37^, and γδ TCRs were observed to bind underneath the antigen-binding platform of MR1^8^, albeit in two orientations that are distinct from the β2-microglobulin dependent mechanism identified here.

More than 25 years ago, the Chien and Mariuzza groups pointed to structural elements in γδ TCRs that are more like immunoglobulins than αβ TCRs. Specifically, their work emphasised the structural resemblance between γδ TCR V domains and antibody V domains^46^, as well as the long CDR3 lengths of γδ TCRs that match more closely to antibodies than to αβ TCRs and potentially offer more conformational flexibility^47^. Our report identifies these and other immunoglobulin-like features of recognition of CD1a. The recognition determinant on CD1a for the CO3 γδ TCR rested principally on one loop, and in this regard the nature of the γδ TCR-CD1a interface, which was rich in aromatic residues and showed high shape complementarity, was reminiscent of antibody–antigen interactions. Further, the two TCR approaches to CD1a for CO3 and CO22 γδ TCRs are distinct from the docking geometries of the two known MR1-binding γδ TCRs^8,37^.

‘End to end’ αβTCR docking topologies with high angles of incidence on MHC I can lead to T cell signalling constraints^48,49^, raising the question of whether sideways docking of γδ TCR on CD1a might be sterically feasible and lead to signalling via the CD3 complex. However, upon stimulation with membrane bound CD1a, both γδTCRs formed signalling competent clusters on the surface of T cells and also induced phosphorylation of Tyr142 on CD3ζ subunit, similar to that of a canonical ‘end to end’ αβ TCR-CD1a engagement. These data rule out a fundamental physical block of clustering or other signalling incompatibility of sideways mode of TCR contact of CD1a.

Instead, the pattern observed here, with intact proximal signalling events, is highly reminiscent of the main population of γδ T cells in mice that manifest constitutive clustering of their TCRs and CD3ζ phosphorylation, but not cytokine production or up-regulation of activation markers. Recent studies show that for mice, such hypo-responsiveness appears to be intrinsic to the γδCD3 complex and is a very broad phenomenon when measured in vivo^41,50^. Thus, mouse TCR hypo-responsiveness is increasingly viewed not as a defect of γδ T cell function, but instead as an adaptive response that confers protection against negative selection^41^, which allows colonisation and survival in the skin and other immunoregulatory niches in response to ‘normality sensing’ of local self-ligands^42^. Experimentally, human γδ T cells are difficult to expand ex vivo^32^, and our TCR clustering and signalling data hint at a possible comparative hypo-responsiveness in the human system. Understanding this phenomenon could explain why human γδ T cells are difficult to capture and study in large numbers ex vivo despite their apparently high numbers in vivo.

Whether unusual γδ TCR docking modalities observed here, and in γδ TCR-MR1 setting, require other factors such as co-stimulatory molecules, remains to be determined. Moreover, how sideways modes of CD1a recognition relate to geometric constraints within the immunological synapse remains unclear but may invoke increased membrane fluidity. Together, CD1a and MR1 reactive γδ TCRs support an evolving picture, whereby the docking geometries underpinning γδ TCR recognition can be vastly different to that of αβ TCR binding modes and one another. Apparently, γδ TCRs are not locked into the familiar vertical approach to antigen presenting molecules, but perhaps resemble antibodies that can approach from any direction and latch onto any feature of an Ag-presenting molecule, or other stress-induced target receptor.

## Methods

### T cells

Human peripheral blood mononuclear cells (PBMCs) were obtained from de-identified, discarded leukoreduction collars provided by the Brigham and Women’s Hospital Specimen Bank, as approved by the Partners Healthcare Institutional Review Board. γδ T cells were enriched using the untouched TCRγ/δ + T cell isolation kit (Miltenyi Biotec).

### Recombinant proteins and tetramers

Human CD1a, CD1b, CD1c, and CD1d monomers were obtained from the National Institute of Health (NIH) tetramer facility. For CD1a-endo, CD1b-endo, CD1c-endo, and CD1d-endo tetramers the monomers were used at 0.2 mg/ml in TBS and tetramerized. Synthetic dideoxymycobactin (3.0 μg) was dried in a 10 mm wide glass tube and dissolved in 6 ml dimethylsulfoxide (DMSO), followed by addition of 90 ml 2% CHAPS in Tris buffered saline and sonication for 1 h at 37 °C. After a short spin, 90 ml was transferred to a plastic tube, followed by addition of 20 μg CD1a monomer and incubation for 2 h at 37 °C. Monomers were tetramerized using streptavidin-APC (Molecular Probes) or streptavidin-PE (Invitrogen).

### Staining and flow cytometry

PBMCs, T cell lines and TCR-transfected cells were stained with tetramers at 2 μg/ml in phosphate buffered saline (PBS) containing 1% bovine serum albumin (BSA) and 0.01% sodium azide. Cells and tetramer were incubated for 10 min at room temperature (RT), followed by addition of unlabelled anti CD3 monoclonal antibody (OKT3) to a final concentration of 2 μg/ml for 10 min at RT, followed by addition of labelled antibodies and another incubation for 10 min at RT, followed by 10 min at 4 °C. Cells were analysed using the BD LSR Fortessa flow cytometer and FlowJo software. Antibodies that were used: CD3-brilliant violet (BV)421 (UCHT1; Biolegend), CD3-Fluorescein isothiocyanate (FITC) (SK7; BD Biosciences), *αβ*TCR-phycoerythrin (PE) or FITC (clone T10B9, BD Biosciences), γδ TCR-PE (B1, BioLegend).

### T cell lines

For generation of T cell lines, cells were stained with CD1a-endo tetramer or antibodies and sorted using a BD FACSAria cell sorter. Expansion of sorted cells was performed by plating cells at 100–700 cells/well in round-bottom 96-well plates containing 2.5 × 10^5^ irradiated allogeneic PBMCs, 5 × 10^4^ irradiated Epstein Barr Virus transformed B cells, and 30 ng/ml anti-CD3 antibody (clone OKT3) per well. The next day human IL-2 or a mix of human IL-2, IL-7, and IL-15 was added to the wells. After 2 weeks, sorting and expansion procedure was repeated as needed.

For CD69 up-regulation and TCR clustering experiments we stably transduced CD1a-restricted TCRs into Jurkat76 cells using the lentiviral transduction system. Briefly, both chains of each TCR (BK6, CO3, CO22) separated by the self-cleaving peptide P2A were cloned in the shuttle vector pLV-EF1a-MCS-IRES-GFP (Biosettia). HEK (Human Embryonic Kidney) 293 T cells were used for lentivirus packaging and the supernatants containing the viral particles were used to transfect Jurkat76 cells. After 4-6 days of culture GFP + Jurkat76 cells were sorted and stained with anti-CD3 antibody to confirm efficient surface expression of the TCRs.

### γδ TCR sequencing and transient transfection

TCR sequences were determined by single cell approach. To each well of a Vapor-Lock (Qiagen)-coated 96 well plate (Eppendorf) a mixture of 0.5 µl 5x reaction buffer, 0.5 μl reverse transcriptase (Iscript, Bio-Rad), and 1.25 µl H_2_O was added per well, with a final concentration of 0.1% 4-(1,1,3,3-tetramethylbutyl)phenyl-polyethylene glycol (Triton X-100). Single cells were sorted into individual wells in this 96 well plate using a FACSAria cell sorter (BD biosciences). The plate was centrifuged at 1400 × *g* at 4 °C for 10 min. For cDNA synthesis, the plate was incubated at 25 °C for 5 min, followed by 42 °C for 30 min, and 80 °C for 5 min. TCR transcripts were amplified in two subsequent, nested polymerase chain reactions (PCRs) using previously published primer sets^51^. The primary reaction consisted of 2.5 μl of the cDNA synthesis reaction mixture as a template, 0.75 units of Thermus aquaticus (Taq) polymerase (Denville), 2.5 µl 10× PCR buffer (Denville), 0.5 µl 10 mM dNTPs, 2.5 pMol of each external TRGV and TRDV primer, 10 pMol antisense TRGC, and 10 pMol antisense TRDC primer in a total volume of 25 ml. The following PCR conditions were used: 95 °C for 5 min, 37 cycles of (95 °C for 45 s, 52 °C for 45 s, 72 °C for 1 min), followed by 1 cycle of 72 °C for 7 min. This reaction mixture was used as a template in two separate secondary PCR reactions. The mixtures are equivalent to the primary PCR except that in one reaction the internal TRGV and TRGC primers were used and in the other reaction the internal TRDV and TRDC primers were used. PCR products were analysed by gel electrophoresis and γ and δ chain PCR products that resulted from the same single cell-containing well were sequenced by Sanger sequencing.

Full length TCR γ and TCR δ chains separated by self-cleaving 2 A peptide were purchased from GENEWIZ and cloned into a (MSCV)-IRES-GFP (pMIG) vector. All plasmids used for transfection were purified using a QIAprep Spin Miniprep Kit (QIAGEN) or NucleoBond Xtra Midi EF kit (Macherey-Nagel). HEK293T cells were co-transfected with pMIG-TCR and pMIG-CD3γ, δ, ε, and ζ using FuGENE-6 (Promega) (51). Expression of TCR and CD3 and binding of tetramer was analysed using the BD LSRFortessa flow cytometer and FlowJo software (version 10.8.1; Treestar & BD Biosciences). For replacement of TCR γ chains, full length TCR γ gene segments were purchased from GENEWIZ and used to replace the γ chain of the CO3 TCR in the plasmid (MSCV)-IRES-GFP (pMIG) using EcoRI and BspEI restriction enzymes.

### Protein production, purification and lipid loading

Human CD1a, CD1a mutants, CD1b, CD1c, CD1d, and CD1 chimeric proteins were expressed in HEK293S cells and purified by nickel-affinity followed by size exclusion chrompatograpy^28^. Proteins used in surface plasmon resonance experiments were biotinylated on their C-terminus using BirA ligase. CD1a used in crystallisation trials was treated with thrombin to remove fos-jun zippers, Avi and His-tags and further de-glycosylated by incubating with endoglycosidase H (NEB) at RT overnight. Biotinylated or thrombin-cleaved CD1a was lipid loaded using lyso-phosphatidylcholine (Avanti 845875), sphingomyelin C42:2 (Avanti 860593), sulfatide (Avanti 131305) and DDM (SYNthesis med chem, Shanghai, China). Each lipid was solubilized in 0.5%CHAPS/TBS buffer at 5–10 mM and was added to CD1a at 10–30 molar excess. The mixture was incubated at RT overnight and the excess lipid/detergent was then removed by size exclusion chromatography run on Superdex200 column (GE Healthcare).

Wild-type extracellular domain of the CO3 γδ TCR was expressed in HEK 293 S cells with 3 C protease-cleavable fos-jun zippers followed by a His-tag at the carboxy terminus and purified via Ni-NTA and size exclusion chromatography. The CO3 and CO22 hybrid γδ/αβ TCRs were expressed as separate α and β chains in BL21 *Escherichia coli* cells as inclusion bodies and solubilised in buffer containing 8 M Urea, 20 mM Tris-HCl pH8.0, 0.2 mM PMSF, 0.5 mM Na-EDTA and 1 mM DTT. The solubilized TCR chains were refolded at a 1:1 ratio in buffer with 5 M urea, 600mM L-Arginine-HCl, 100 mM Tris-HCl pH8.5, 2 mM Na-EDTA, 0.2 mM PMSF, 0.5 mM oxidised glutathione and 5 mM reduced glutathione overnight at 4 °C. The refolded samples were then dialysed three times against 10 mM Tris-HCl pH8.5 over 24 h. CO3 γδ TCR and CO22 γδ TCR were both purified by DEAE cellulose and hydrophobic interaction followed by a size exclusion chromatography for CO3 γδ TCR and anion exchange Mono Q for CO22 γδ TCR. The purity and quality of protein were analysed by SDS-PAGE.

### Surface plasmon resonance

Biotinylated monomers of CD1 variants were coupled onto SA Sensor Chips (Cytiva) to approximately 2500 response units per flow cell. Affinity measurements were carried out at 25 °C in 20 mM Tris pH8, 150 mM NaCl, 0.5% BSA buffer and consisted of serial injections of the analyte TCR to a maximum concentration of 50–100 μM. The data were analysed using Scrubber 2. The dissociation constant was calculated by fitting the binding response to a one site binding model in GraphPad.

### Crystallisation, data collection and structure determination

CO3 γδ TCR alone crystallised in 0.1 M MES pH6, 0.15 ammonium sulfate, 15% PEG4000 and diffracted to 2.0 Å at the MX2 beamline (Australian Synchrotron). Upon data processing in XDS and CCP4, the structure was solved by molecular replacement in phenix.phaser using BK6 αβ TCR structure (PDB 4X6B) as search model^52–54^. The complete hybrid TCR model was generated using the AutoBuild module from Phenix and was further refined by combining automated and manual refinement cycles in phenix.refine and Coot, respectively, until the final R_work_/R_free_ of 20/25% was reached^55,56^. CO3 and CD1a-endo were mixed at 1:1 molar ratio and incubated at 4 C overnight to allow complex formation. Ternary complex crystals appeared after 3–4 days in 20% PEG 3350, 0.2 M ammonium sulphate or 0.1 M MES pH 6, 0.15 M ammonium sulfate, 15% PEG 4000. Crystals were cryoprotected in the well solution supplemented with 20% glycerol or 10% ethylene glycol and flash frozen in liquid nitrogen. CO3 γδ TCR -CD1a-endo crystals diffracted to 3.2 Å at the MX2 beamline (Australian Synchrotron). The crystals of CO3 γδ TCR -CD1a-sulfatide and CO3 γδ TCR -CD1a-DDM were obtained in the same condition using CO3-CD1a-endo crystals as seeds and were diffracted at the MX2 beamline (Australian Synchrotron) to 2.7 Å and 3.0 Å respectively. Data processing was performed in XDS and CCP4. The structure of CO3 γδ TCR -CD1a-endo was solved by molecular replacement using a CD1a binary structure (PDB 7KP1) and the CO3 γδ TCR alone as search models. The resulting ternary model was used in the molecular replacement search for CO3 γδ TCR-CD1a-sulfatide and CO3 γδ TCR-CD1a-DDM complexes. The constant domains of CO3 TCR were highly disordered in the CO3-CD1a-endo complex and could be only partially traced, however, the electron density map was significantly improved in the CO3-CD1a-DDM and CO3-CD1a-sulfatide complexes allowing for both constant domains to be built. The final models were obtained by refinement cycles in phenix.refine and Coot until the R_work_/R_free_ values reached 25/29, 23/27, and 22/27% for CO3-CD1a-endo, CO3-CD1a-sulfatide and CO3-CD1a-DDM respectively. The quality of the data was validated by Research Collaboratory for Structural Bioinformatics Protein Data Bank Data Validation and Deposition Services website. Contact residues and complementarity score were calculated in CCP4, buried surface area analyses and graphics were undertaken in UCSF Chimera^57^.

### T cells activation assays and flow cytometry

Approximately 1 × 10^5^ Jurkat76 cells expressing the TCRs of interest were seeded per well of a 96-well round bottom plate. To check the up-regulation of CD69, the Jurkat cells were co-cultured for 16 h with CD3/CD28 dynabeads (Gibco) or CellTrace Violet (cat. no. C34571, ThermoFisher Scientific, MA, USA) labelled parental K562 (CD1a^-^) or CD1a-expressing K562 cells at a 1:1 T cell: APC/bead ratio. K562 cells were labelled with CellTrace Violet as per the manufacturer’s instructions prior to the co-culture. After 16 h, cells were harvested and washed twice in PBS. Cells were then incubated with 1:500 diluted Zombie Aqua Fixable Viability Kit (cat. no. 423101, BioLegend, CA, USA) for 10 minutes (min) at room temperature (RT). Cells were washed in FACS buffer (i.e., PBS supplemented with 2% foetal calf serum (FCS) and 0.04% Sodium Azide (both Sigma-Aldrich, MO, USA) and stained with anti-human CD3-APC (1:100 dilution; clone SP34-2, cat. no. 557597) and CD69-BV650 (1:50 dilution; clone FN50, cat. no. 563835, both BD Biosciences, CA, USA) for 15 min on ice. After washing in FACS buffer, cells were fixed using IC Fixation Buffer (ThermoFisher Scientific) for 10 min at RT, washed and acquired on a BDFortessa X20 (BD Biosciences). Resulting data was analysed in FlowJo (version 10.8.1; Treestar & BD Biosciences) and a representative gating strategy is given in Supplementary Fig. S7.

### Preparation of supported lipid bilayer (SLB)

Glass coverslips of 0.17 mm thickness were thoroughly cleaned with 1 M KOH and rinsed with Milli-Q water and placed in 100% ethanol prior to drying inside a fume hood. Following plasma cleaning, coverslips were adhered to eight-well silicon chambers (ibidi, #80841). SLB was prepared by vesicle extrusion of 1 mg/ml liposome solution^43^. The lipid composition of liposomes include 96.5% DOPC (1,2-dioleoyl-sn-glycero-3- phosphocholine), 2% DGS-NTA(Ni) (1,2-dioleoyl-sn- glycero-3-[(N-(5-amino-1-carboxypentyl)iminodiacetic acid)succinyl] (nickel salt)), 1% Biotinyl-Cap-PE (1,2-dioleoyl-sn-glycero-3-phosphoethanolamine-N-(cap biotinyl) (sodium salt)), and 0.5% PEG5000-PE (1,2-distearoyl-sn-glycero-3- phosphoethanolamine-N-[methoxy(poly- ethylene glycol)−5000] (ammonium salt) (mol%; all available from Avanti Polar Lipids (DOPC, 850375 C), (DGS-NTA(Ni), 790404 C), (Biotinyl-Cap-PE, 870273 C), (PEG5000-PE, 880220 C). Extruded liposomes were added to eight-well chambers at a ratio of 1:5 with Milli-Q water (10 mM CaCl_2_) and incubated for 30 min at RT before gently rinsing with TBS repeatedly. By retaining ~200 µl of TBS in each well, disruption to SLB was minimised during washing steps. Fluorescence recovery after photobleaching (FRAP) was used to examine the lateral mobility of freshly prepared SLB by adding fluorescent streptavidin (Invitrogen, #S11223)^43^. Excess Ca^2+^ ions on SLB were removed with 0.5 mM EDTA, followed by gentle rinsing with Milli-Q water. The functionalised NTA groups in DGS-NTA(Ni) lipids were recharged by adding 1 mM NiCl_2_ solution to SLB for 15 min. Excess Ni^2+^ ions were removed by repeated washing with TBS.

### Stimulation and immunostaining of T cells on SLB

The functionalised biotin groups on SLB were coupled to 100 µg/ml streptavidin (Invitrogen, #434301) followed by a second coupling to 500 ng/ml biotinylated CD1a-endo or 500 ng/ml biotinylated anti-CD3 (Invitrogen, #13-0037-82) and anti-CD28 monoclonal antibodies (Invitrogen, #13-0289-82). NTA functionalised lipids were coupled with 200 ng/ml of His-tagged ICAM-1 (Sino Biological, # 10346-H08H). SLB was repeatedly rinsed with TBS to remove excess unbound proteins. Before adding Jurkat T cells, SLB was incubated with warm RPMI culture medium (37 °C) for 30 min. T cells were activated on SLB for 5 min at 37 °C, followed by immediate cell fixation with 4% paraformaldehyde (vol/vol) in PBS for 15 min at RT and then rinsed with PBS. T cells were permeabilised with 0.1% Triton X-100 (vol/vol) (Sigma-Aldrich) for 15 min and then rinsed with PBS. Cells were then blocked with 5% bovine serum albumin in PBS and immunostained with anti-CD3ε-Alexa Fluor 647 (BioLegend, #300416, Clone UCHT1) and anti-pCD3ζ-Alexa Fluor 568 (BD Biosciences, #558402) antibodies (1:300 dilution) for 1 h at RT and rinsed with PBS. A post-fixation step was carried out using 4% paraformaldehyde (vol/vol) in PBS for 15 min. Finally, 0.1 µm TetraSpeck microspheres (Invitrogen, #T7279) were embedded onto the lipid bilayer.

### Single-molecule imaging with direct stochastic optical reconstruction microscopy (*d*STORM)

Imaging buffer consisting of TN buffer (50 mM Tris-HCl pH 8.0, 10 mM NaCl), oxygen scavenger system GLOX [0.5 mg/ml glucose oxidase (Sigma- Aldrich, #G2133); 40 mg/ml catalase (Sigma- Aldrich, #C-100); and 10% w/v glucose], and 10 mM 2-aminoethanethiol (MEA; Sigma- Aldrich, #M6500) was used for single-molecule imaging with *d*STORM. Image sequences for *d*STORM were acquired on a total internal reflection fluorescence (TIRF) microscope (Nanoimager by ONI) equipped with a 100× (1.4NA) oil immersion objective, XYZ closed-loop piezo stage, and lasers 405 nm (150 mW), 473 nm (1 W), 561 nm (1 W) and 640 nm (1 W) at 30 °C. Time series of 10,000 frames were acquired per sample, per channel (640 or 561 nm laser channel) with an exposure time of 30 ms, at near-TIRF angle of 54°. For dual-colour acquisition, higher wavelength channel (640 nm laser for Alexa Fluor 647) was acquired first, followed by the channel with shorter wavelength (561 nm laser for Alexa Fluor 568) using a sCMOS camera (ORCA Flash 4, Hamamatsu). Image processing, including fiducial markers-based drift correction, two-channel alignment, and generation of x-y particle coordinates for each localisation was carried out by ONI proprietary software (version 1.16).

### Ripley’s K analysis

To perform Ripley’s K analysis on single-molecule images^43^ we used the linearised form of Ripley’s (K) defined as L(*r*) − *r*, where *r* is the spatial scale radius. While in complete spatial randomness L(*r*) – *r* = 0, a positive or negative value for L(*r*) – *r* can indicate clustered or dispersed localisations respectively^58^. For each localisation, Ripley’s (K) calculates the number of neighbouring localisations within a given radius (*r*) corrected by the total density of localisations. The start (0 nm), end (100 nm), and step size (10 nm) for *r* in the algorithm were user defined.

### Cluster analysis of single-molecule images

For quantification of cluster parameters in single-molecule images, we used a custom-build algorithm^43^ that utilises a density-based spatial clustering with noise (DBSCAN) analysis implemented in MATLAB to quantify individual clusters. Here, we pre-determine the minimum number of neighbours (minimum points = 3) and the radius which they occupy (*r* = 20 nm). The combined cluster detection and colocalisation (Clus-DoC) analysis was performed to quantify both spatial distribution and the degree of colocalization of two proteins/receptors^59^. This analysis relies on generating density gradients for each individual localisation by calculating the number of molecules captured from both channels with increasing circle radius (*r* = 20 nm). These density gradients are then normalised to the density at the maximum radius respectively for channel 1 and channel 2. The resulting two types of distributions generated for each channel were then compared by calculating the rank correlation coefficient using Spearman correlation where the local coefficient was measured by a value proportional to the distance of the nearest neighbour. Accordingly, each localisation was assigned with a DoC score ranging from +1 (indicate colocalization) to −1 (indicate segregation) with 0 indicating random distribution. As previously described^59^, the threshold for DoC is a user-defined variable and can be optimised to different experimental conditions. Hence, the DoC threshold for colocalisation was set to DoC ≥0.1, above which the values represent colocalization events captured between the two channels.

### Statistical analyses

When comparing multiple groups, the statistical analysis was performed using one-way ANOVA in GraphPad Prism software (version 9.31). Statistical significance reported by P-values indicated as ns (no significance); *P ≤ 0.05; **P ≤ 0.01; ***P ≤ 0.001; ****P ≤ 0.0001. Error bars represent the SEM.

### Reporting summary

Further information on research design is available in the Nature Research Reporting Summary linked to this article.

## Supplementary information

Supplementary Information

Reporting Summary

## Data Availability

The structure factors and PDB coordinates of the crystal structures generated in this study have been deposited in the RCSB Protein Data Bank under the following accession codes: 7RYL, (CO3 binary); 7RYM, (CO3-CD1a-endo); 7RYN, (CO3-CD1a-sulfatide); 7RYO, (CO3-CD1a-DDM). The structural data used for molecular replacement in this study are available in the RCSB Protein Data Bank under accession codes 4X6B and 7KP1. The surface plasmon resonance binding data generated in this study are provided in the Source Data file. T cell activation data and single cell image analyses are provided in the Source Data file. Source data are provided with this paper.

## Source data

Source Data

## Code availability

The link to custom-build cluster analysis algorithm used in this study is available at GitHub repository link (https://github.com/PRNicovich/ClusDoC) which was previously published^59^.

## References

[1] Morrissey KA (2021). The molecular assembly of the marsupial gammamu T cell receptor defines a third T cell lineage. Science.

[2] Rossjohn J (2015). T cell antigen receptor recognition of antigen-presenting molecules. Annu. Rev. Immunol..

[3] Borg NA (2007). CD1d-lipid-antigen recognition by the semi-invariant NKT T-cell receptor. Nature.

[4] Garboczi DN (1996). Structure of the complex between human T-cell receptor, viral peptide and HLA-A2. Nature.

[5] Garcia, K. C. et al. An alphabeta T cell receptor structure at 2.5 A and its orientation in the TCR-MHC complex. Science (New York, N.Y.) 274, 209–219 (1996). 10.1126/science.274.5285.209 8824178

[6] Willcox BE, Willcox CR (2019). γδ TCR ligands: the quest to solve a 500-million-year-old mystery. Nat. Immunol..

[7] Uldrich AP (2013). CD1d-lipid antigen recognition by the gammadelta TCR. Nat. Immunol..

[8] Le Nours J (2019). A class of gammadelta T cell receptors recognize the underside of the antigen-presenting molecule MR1. Science.

[9] Adams EJ, Luoma AM (2013). The adaptable major histocompatibility complex (MHC) fold: structure and function of nonclassical and MHC class I–like molecules. Annu. Rev. Immunol..

[10] Harly, C. et al. Human γδ T cell sensing of AMPK-dependent metabolic tumor reprogramming through TCR recognition of EphA2. *Sci. Immunol.* **6** (2021). 10.1126/sciimmunol.aba9010 34330813

[11] Willcox CR (2012). Cytomegalovirus and tumor stress surveillance by binding of a human [gamma][delta] T cell antigen receptor to endothelial protein C receptor. Nat. Immunol..

[12] Nicolai S (2020). Human T cell response to CD1a and contact dermatitis allergens in botanical extracts and commercial skin care products. Sci. Immunol..

[13] Kim JH (2016). CD1a on Langerhans cells controls inflammatory skin disease. Nat. Immunol..

[14] Van Rhijn I, Godfrey DI, Rossjohn J, Moody DB (2015). Lipid and small-molecule display by CD1 and MR1. Nat. Rev. Immunol..

[15] Kasmar A, Van Rhijn I, Moody DB (2009). The evolved functions of CD1 during infection. Curr. Opin. Immunol..

[16] Brigl M, Brenner MB (2004). CD1: antigen presentation and T cell function. Annu. Rev. Immunol..

[17] Gadola SD (2002). Structure of human CD1b with bound ligands at 2.3 A, a maze for alkyl chains. Nat. Immunol..

[18] Giabbai B (2005). Crystal structure of mouse CD1d bound to the self ligand phosphatidylcholine: a molecular basis for NKT cell activation. J. Immunol..

[19] Scharf L (2010). The 2.5 A structure of CD1c in complex with a mycobacterial lipid reveals an open groove ideally suited for diverse antigen presentation. Immunity.

[20] Zajonc DM, Elsliger MA, Teyton L, Wilson IA (2003). Crystal structure of CD1a in complex with a sulfatide self antigen at a resolution of 2.15 A. Nat. Immunol..

[21] Zajonc DM, Elsliger MA, Teyton L, Wilson IA (2003). Crystal structure of CD1a in complex with a sulfatide self antigen at a resolution of 2.15 angstrom. Nat. Immunol..

[22] Agea E (2005). Human CD1-restricted T cell recognition of lipids from pollens. J. Exp. Med..

[23] Reijneveld JF (2020). Human γδ T cells recognize CD1b by two distinct mechanisms. Proc. Natl Acad. Sci. USA.

[24] Bai L (2012). The majority of CD1d-sulfatide-specific T cells in human blood use a semiinvariant Vdelta1 TCR. Eur. J. Immunol..

[25] Roy S (2016). Molecular Analysis of Lipid-Reactive Vdelta1 gammadelta T Cells Identified by CD1c Tetramers. J. Immunol..

[26] Luoma AM (2013). Crystal structure of Vdelta1 T cell receptor in complex with CD1d-sulfatide shows MHC-like recognition of a self-lipid by human gammadelta T cells. Immunity.

[27] Altman, J. D. et al. Phenotypic analysis of antigen-specific T lymphocytes. Science (New York, N.Y.) 274, 94–96 (1996). 21690331

[28] Cotton RN (2021). Human skin is colonized by T cells that recognize CD1a independently of lipid. J. Clin. Invest..

[29] Cotton RN (2021). CD1a selectively captures endogenous cellular lipids that broadly block T cell response. J. Exp. Med..

[30] Birkinshaw RW (2015). alphabeta T cell antigen receptor recognition of CD1a presenting self lipid ligands. Nat. Immunol..

[31] de Jong A (2014). CD1a-autoreactive T cells recognize natural skin oils that function as headless antigens. Nat. Immunol..

[32] Spada FM (2000). Self-recognition of CD1 by gamma/delta T cells: implications for innate immunity. J. Exp. Med..

[33] Adams EJ, Chien YH, Garcia KC (2005). Structure of a gammadelta T cell receptor in complex with the nonclassical MHC T22. Science.

[34] Reijneveld JF (2020). Human gammadelta T cells recognize CD1b by two distinct mechanisms. Proc. Natl Acad. Sci. USA.

[35] Moody DB (2004). T cell activation by lipopeptide antigens. Science.

[36] Zajonc DM (2005). Molecular mechanism of lipopeptide presentation by CD1a. Immunity.

[37] Rice MT (2021). Recognition of the antigen-presenting molecule MR1 by a Vdelta3(+) gammadelta T cell receptor. Proc. Natl Acad. Sci. USA.

[38] Willcox CR (2019). Butyrophilin-like 3 directly binds a human Vγ4(+) T cell receptor using a modality distinct from clonally-restricted antigen. Immunity.

[39] La Gruta NL, Gras S, Daley SR, Thomas PG, Rossjohn J (2018). Understanding the drivers of MHC restriction of T cell receptors. Nat. Rev. Immunol..

[40] Young DC (2009). Synthesis of dideoxymycobactin antigens presented by CD1a reveals T cell fine specificity for natural lipopeptide structures. J. Biol. Chem..

[41] Wencker M (2014). Innate-like T cells straddle innate and adaptive immunity by altering antigen-receptor responsiveness. Nat. Immunol..

[42] McKenzie DR (2022). Normality sensing licenses local T cells for innate-like tissue surveillance. Nat. Immunol..

[43] Pageon SV (2016). Functional role of T-cell receptor nanoclusters in signal initiation and antigen discrimination. Proc. Natl Acad. Sci. USA.

[44] Wun KS (2018). T cell autoreactivity directed toward CD1c itself rather than toward carried self lipids. Nat. Immunol..

[45] Zeng X (2012). γδ T cells recognize a microbial encoded B cell antigen to initiate a rapid antigen-specific interleukin-17 response. Immunity.

[46] Li H (1998). Structure of the Vdelta domain of a human gammadelta T-cell antigen receptor. Nature.

[47] Rock EP, Sibbald PR, Davis MM, Chien YH (1994). CDR3 length in antigen-specific immune receptors. J. Exp. Med..

[48] Zareie P (2021). Canonical T cell receptor docking on peptide-MHC is essential for T cell signaling. Science.

[49] Singh NK (2020). An engineered T cell receptor variant realizes the limits of functional binding modes. Biochemistry.

[50] Chodaczek G, Papanna V, Zal MA, Zal T (2012). Body-barrier surveillance by epidermal gammadelta TCRs. Nat. Immunol..

[51] Guo XZ (2016). Rapid cloning, expression, and functional characterization of paired αβ and γδ T-cell receptor chains from single-cell analysis. Mol. Ther. Methods Clin. Dev..

[52] Kabsch W (2010). Xds. Acta Crystallogr D. Biol. Crystallogr..

[53] McCoy AJ (2007). Phaser crystallographic software. J. Appl. Crystallogr..

[54] Winn MD (2011). Overview of the CCP4 suite and current developments. Acta Crystallogr. D. Biol. Crystallogr..

[55] Afonine PV (2012). Towards automated crystallographic structure refinement with phenix.refine. Acta Crystallogr. D. Biol. Crystallogr..

[56] Emsley P, Lohkamp B, Scott WG, Cowtan K (2010). Features and development of Coot. Acta Crystallogr. D. Biol. Crystallogr..

[57] Pettersen EF (2004). UCSF Chimera–a visualization system for exploratory research and analysis. J. Comput. Chem..

[58] Ripley BD (1979). Tests of ‘Randomness’ for spatial point patterns. J. R. Stat. Soc. Ser. B (Methodol.).

[59] Pageon SV, Nicovich PR, Mollazade M, Tabarin T, Gaus K (2016). Clus-DoC: a combined cluster detection and colocalization analysis for single-molecule localization microscopy data. Mol. Biol. Cell.

