# Supplementary material for: Atypical sideways recognition of CD1a by autoreactive γδ T cell receptors
Source: Nat Commun. 2022 Jul 5;13:3872. doi: 10.1038/s41467-022-31443-9 (PMC9256601; doi:10.1038/s41467-022-31443-9)
Supplement: Supplementary file 1 — Supplementary Information [file 41467_2022_31443_MOESM1_ESM.pdf]

Supplementary Information

Atypical sideways recognition of CD1a by autoreactive γδ T cell receptors

Marcin Wegrecki[1], Tonatiuh A. Ocampo[2], Sachith D. Gunasinghe[1,3], Anouk von Borstel[1], Shin Yi Tin[1], Josephine F. Reijneveld[2,4], Thinh-Phat Cao[1], Benjamin S. Gully[1], Jérôme Le Nours[1], D. Branch Moody[2*], Ildiko van Rhijn[2,4*] & Jamie Rossjohn[1,5*]

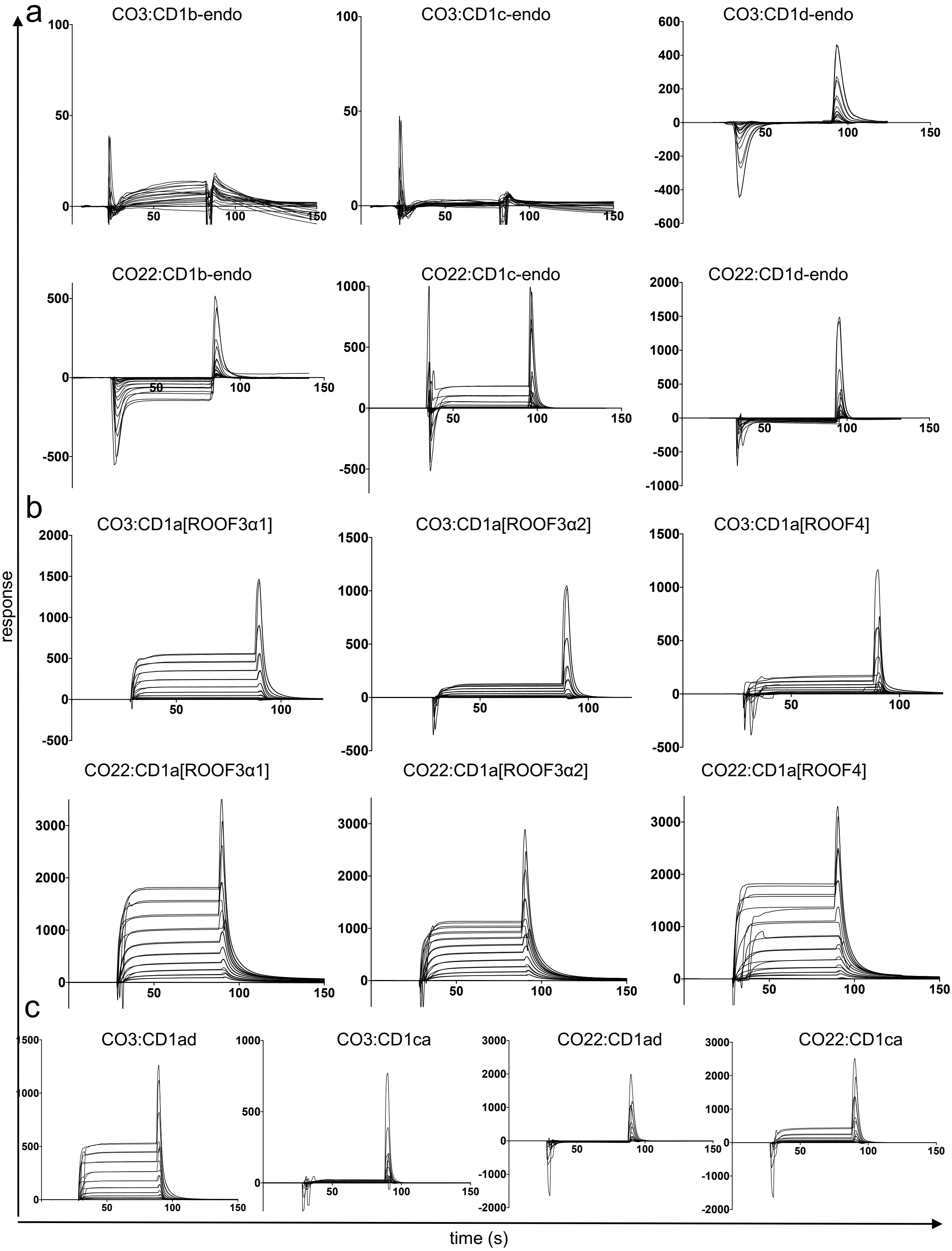

Supplementary Figure S1. Surface plasmon resonance sensorgrams corresponding to the serial injections of CO3 and CO22 γδTCRs injected over a flow cell containing different human CD1 isoforms (a), multiple mutants across the A' roof of human CD1a (b), or CD1-α3 domain chimeras (c). Source data are provided as a Source Data file.

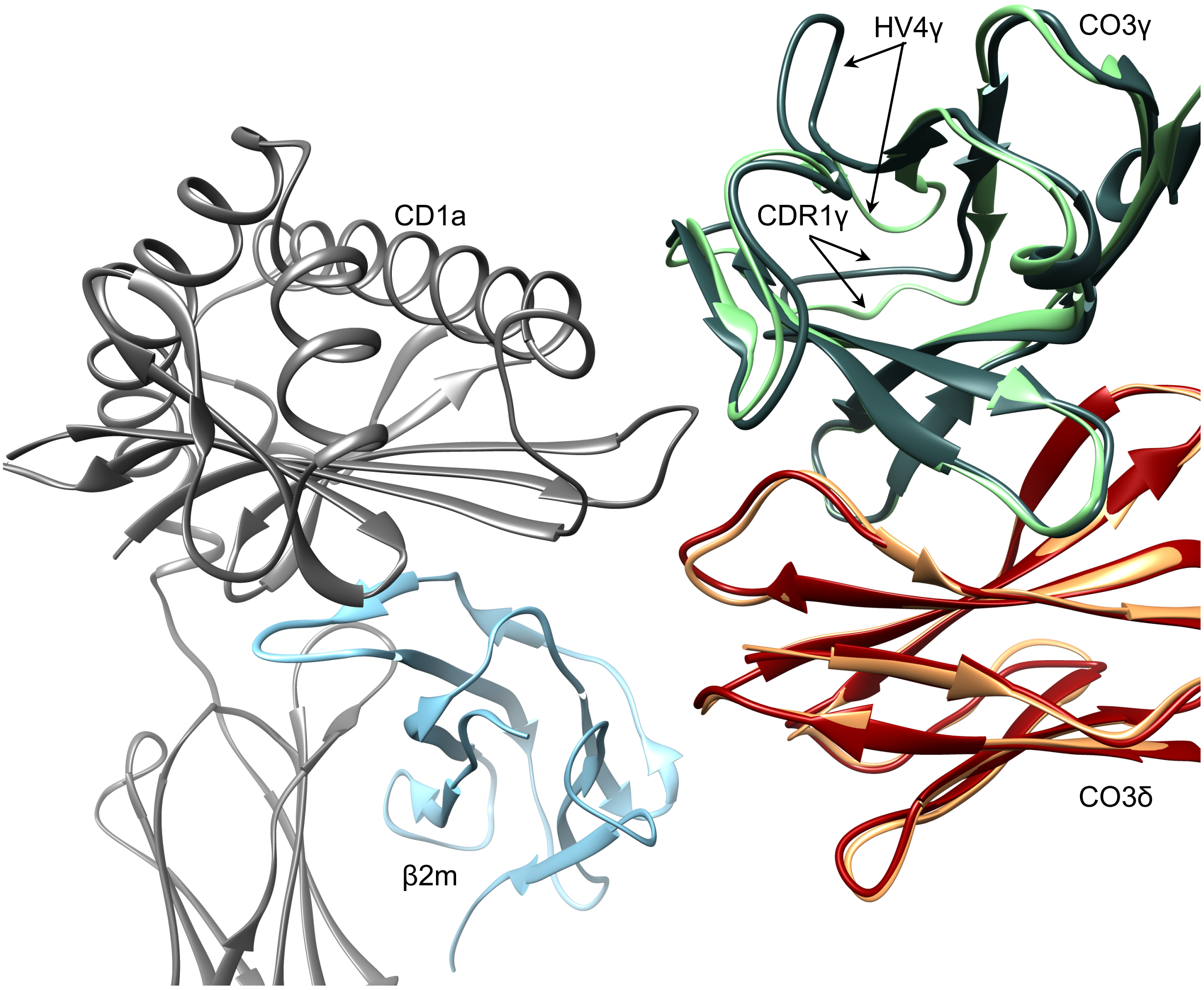

Supplementary Figure S2. Comparison of the binary crystal structure of CO3 γδTCR (γ chain dark green, δ chain dark red) and the structure of CO3 bound to CD1a (γ chain green, δ chain orange) shows no major structural rearrangements within the area of the TCR involved in the complex formation. The most prominent conformational differences between CD1a-bound and TCR binary structures reside within CDR1γ loop and HV4 region of CO3 γδTCR (arrows) that do not make contacts with CD1a.

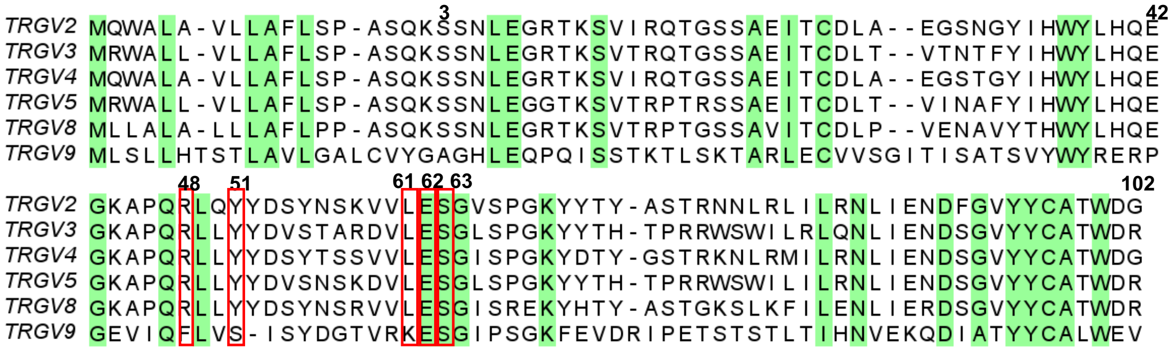

Supplementary Figure S3. Sequence alignment of the functional human TRGV gene products. Fully conserved residues are highlighted in green. The position and numbering of the FRγ residues in CO3 γδTCR interacting with CD1a-sulfatide are shown in red boxes.

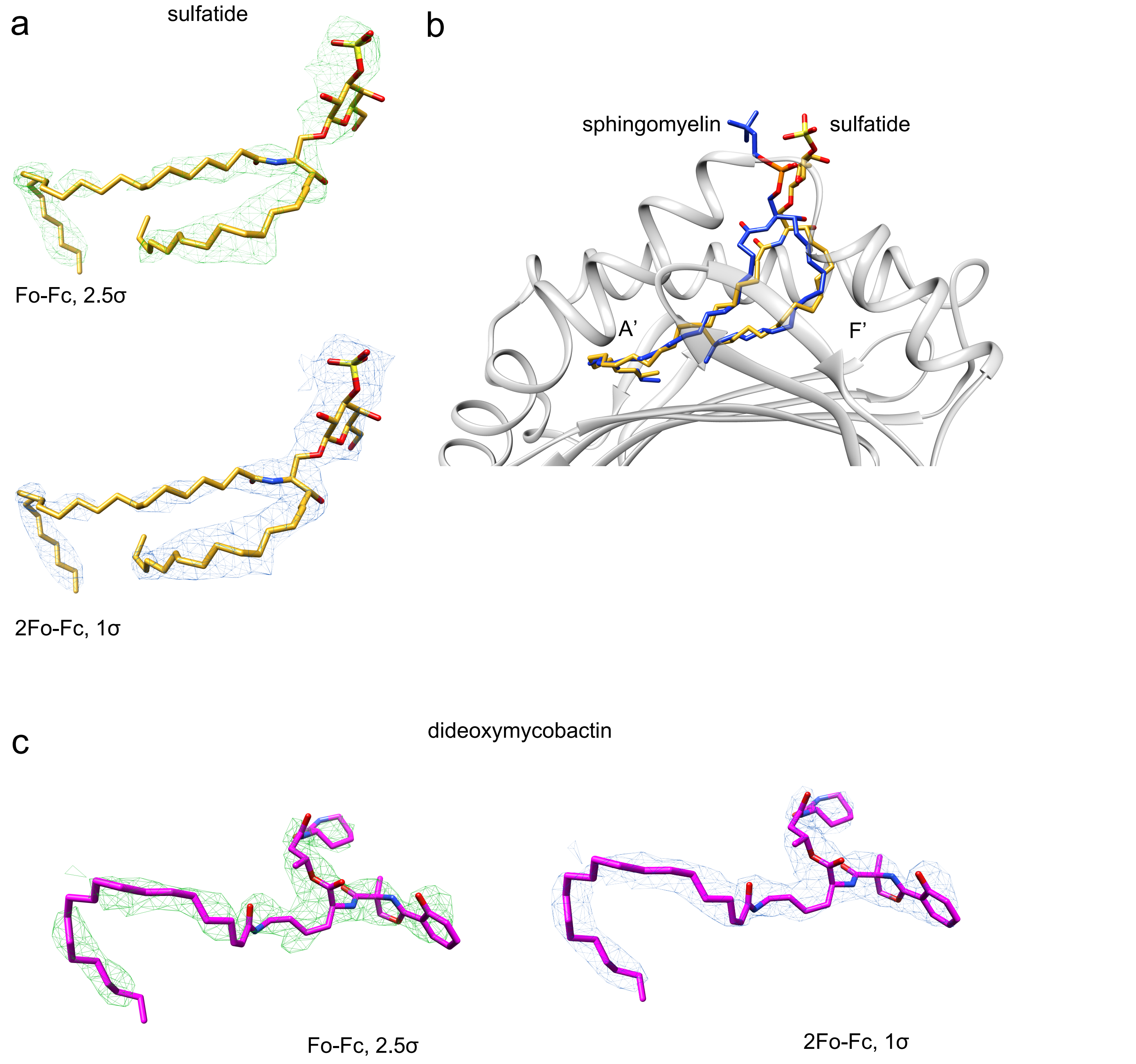

Supplementary Figure S4. Electron density maps for lipid ligand in the cleft of CD1a **a)** Unbiased (Fo-Fc at 2.5 σ, green) and refined (2Fo-Fc at 1.0 σ, blue) electron density maps for 24:1 sulfatide (yellow) in the cleft of CD1a from the CO3:CD1a-sulfatide ternary complex structures. **b)** Superimposition of 42:2 sphingomyelin (navy blue) and sulfatide (yellow) in the binding cleft of CD1a (grey) showing similarities in relative orientation of the lipid tails and the headgroup of the lipid antigen. **c)** Unbiased (Fo-Fc at 2.5 σ, green) and refined (2Fo-Fc at 1.0 σ, blue) electron density maps for dideoxymycobactin (magenta) in the cleft of CD1a in the CO3:CD1a-dideoxymycobactin ternary complex structures.

Supplementary Fig. S5 Wegrecki et al

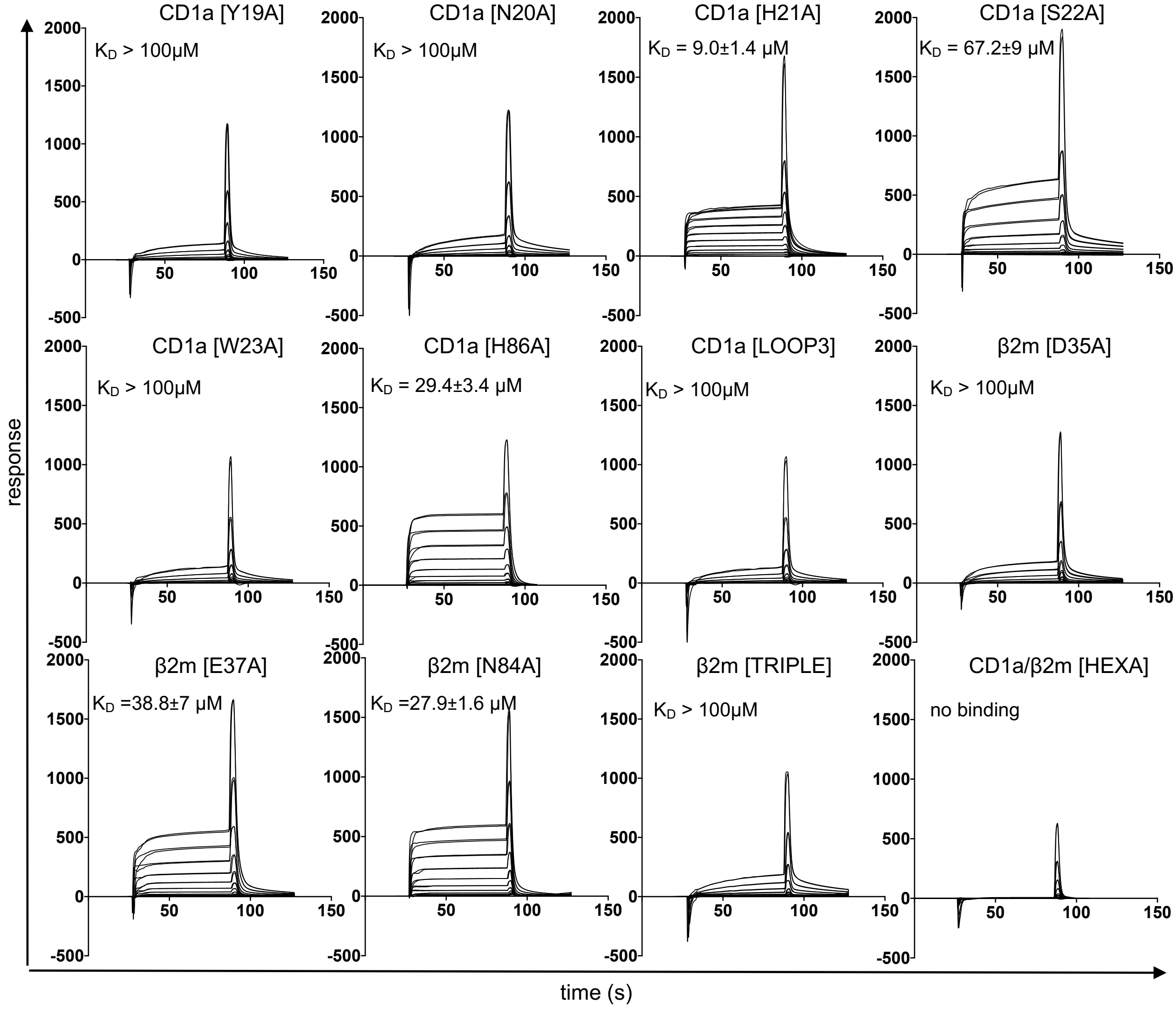

Supplementary Figure S5. SPR sensorgrams for serial injections of CO3 γδTCR from 0 to 100 μM over a flow cell containing the variant of CD1a/β2m indicated for each panel. The sensorgrams represent one experiment, in which two independent injections of each TCR sample were performed. The K_D was calculated from two independent experiments. Source data are provided as a Source Data file.

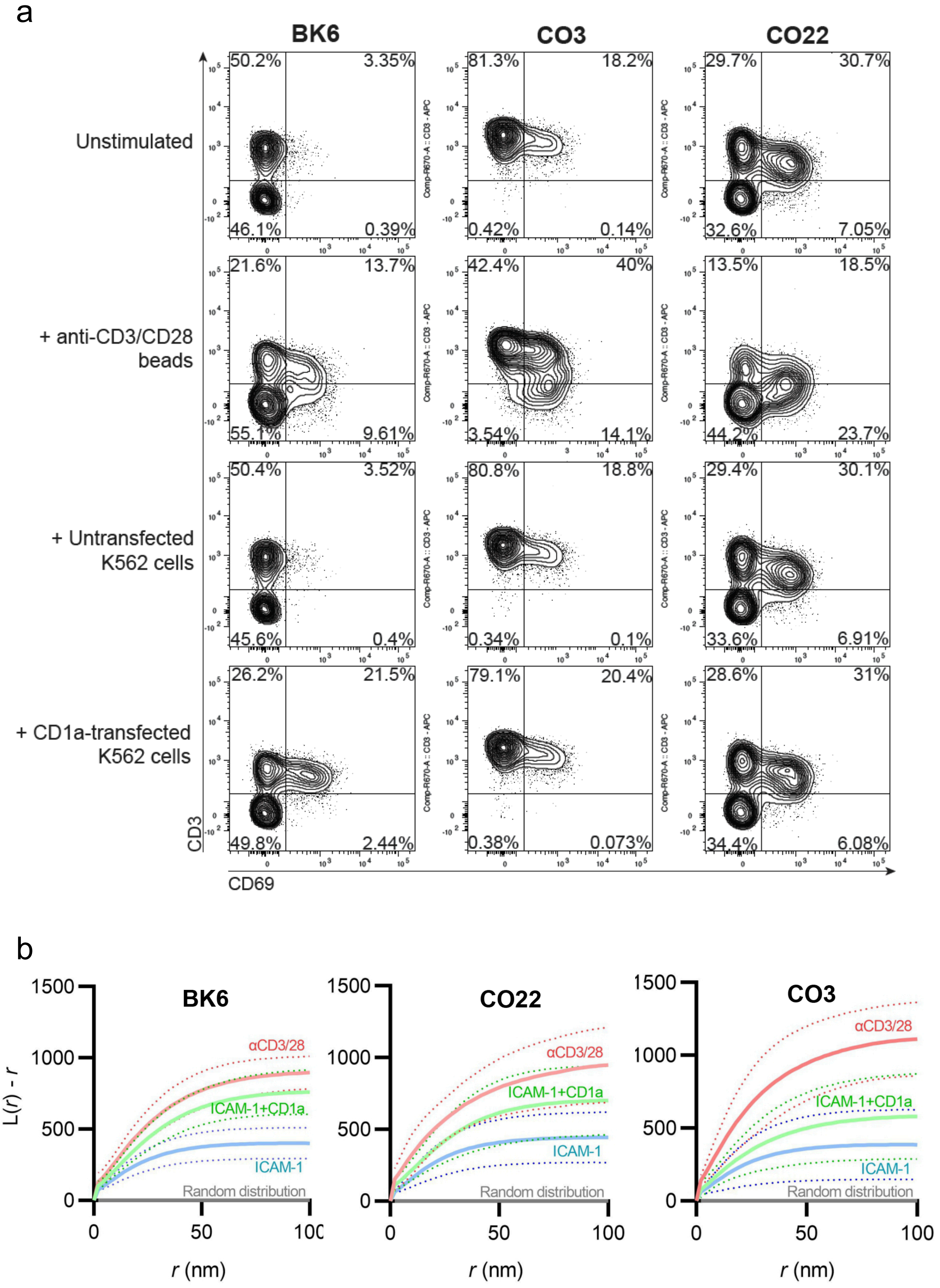

Supplementary Figure S6. Functional analysis of Jurkat76 cells expressing CD1a-restricted TCRs. **a)** The level of CD69 expression on transduced Jurkat76 cell lines was analysed by flow cytometry upon stimulation of cells with either CD3/CD28 dynabeads, or co-culture with $CD1a^-$ K562 cells or K562 cells stably expressing CD1a. The cells were gated as shown in **Supplementary Fig. S7**. Each activation assay was performed at least twice in two independent experiments. **b)** Ripley's K analysis of CD3ε clustering [L(*r*)-*r*] against radii (*r*). Complete spatial randomness is shown as a solid grey line where L(*r*)-*r* = 0. Positive L(*r*)-*r* values indicate molecular clustering (ICAM-1 in blue, ICAM1+CD1a in green, and anti-CD3/CD28 in red) relative to the random distribution, shown as the mean (solid line) ± 95% confidence interval (dashed lines). Dotted lines indicate ± SEM. Experiments were performed in triplicates. Source data are provided as a Source Data file.

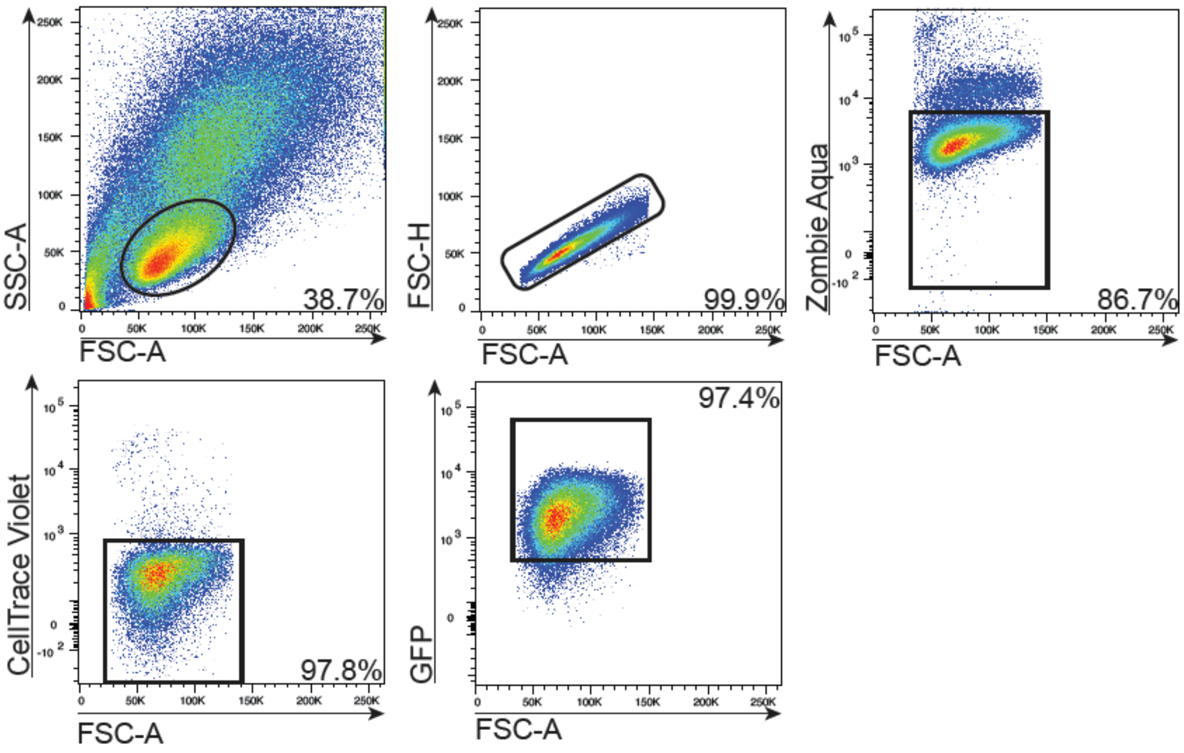

Supplementary Figure S7. Gating strategy used in the CD3/CD69 analysis of Jurkat76 cell lines stably expressing CD1a-restricted αβTCR (BK6) or γδTCRs (CO3 and CO22) shown in **Supplementary Fig. S6a**.

Supplementary Table 1. Binding affinity values calculated using surface plasmon resonance between γδTCRs and CD1a carrying endogenous or defined lipid antigens.

	CO3 K_D (μM)	CO22 K_D (μM)
CD1a-endogenous (endo)	23.6±3.1	15.5±0.2
CD1a-lyso-phosphatidylcholine (LPC)	16.3±3.0	11.8±5.2
CD1a-C42:2 sphingomyelin (42:2 SM)	15.2±0.2	10.3±5.7
CD1a-sulfatide (SLF)	21.9±4.8	13.1±0.7
CD1a-dideoxymycobactin (DDM)	8.7±1	11.4±0.8

Supplementary Table 2. Data collection and refinement statistics on CD1a-CO3 γδTCR complexes.

	CO3:CD1a-endo	CO3:CD1a-sulfatide	CO3:CD1a-DDM	CO3 γδTCR binary
**Data collection statistics**				
Temperature (K)	100	100	100	100
Wavelength (Å)	0.954	0.954	0.954	0.954
Resolution Range (Å)	38.4-3.2 (3.3-3.2)	42.9-2.7 (2.8-2.7)	43.9-3.0 (3.2-3.0)	45.5-2.0 (2.1-2.0)
Space group	C2	C2	C2	$P\ 2_1\ 2_1\ 2_1$
Unit cell				
a, b, c (Å)	237.1, 42.5, 121.6	234.7, 42.2, 123.1	234.9, 42.3, 124.7	51.0, 76.4, 113.2
α, β, γ (°)	90.0, 116.1, 90	90.0, 114.3, 90.0	90, 113.4, 90	90.0, 90.0, 90.0
Total reflections	127129 (13075)	352643 (46120)	158475 (26171)	208255 (15218)
Unique reflections	18546 (1832)	31003 (4097)	23043 (3638)	30668 (2241)
Multiplicity	6.9 (7.1)	11.4 (11.3)	6.9 (7.2)	6.8 (6.8)
Completeness (%)	99.6 (99.5)	99.9 (99.6)	99.7 (99.6)	100 (100)
Mean I/σ_I	12.4 (2.4)	14.2 (2.9)	6.5 (1.6)	10.3 (1.6)
$R_{p.i.m.}$[1] (%)	7.1 (36.3)	3.1 (25.5)	10 (57.9)	5.8 (74.3)
Wilson B-factor ($Å^2$)	81.3	60.4	62.4	30.7
**Refinement statistics**				
R_{work}[2] (%)	24.7	23.2	22.2	19.9
R_{free}[3] (%)	28.6	26.9	26.7	24.7
Non-hydrogen atoms	5542	6091	6288	3746
macromolecules	5530	5961	6215	3441
ligands	12	116	73	14
solvent	0	14	0	291
Protein residues	732	781	806	432
rmsd bonds (Å)	0.002	0.002	0.002	0.003
rmsd angles (°)	0.63	0.60	0.60	0.64
Ramachandran				
favored (%)	93.5	96.1	92.3	95.8
allowed (%)	6.3	3.9	6.5	3.8
outliers (%)	0.2	0.0	0.5	0.4
Average B-factor ($Å^2$)	128	100	79	40
protein	128	100	79	40
ligand	99	91	67	55
water	-	63	-	40

Statistics for the highest-resolution shell are shown in parentheses.

[1] $R_{p.i.m} = \Sigma_{hkl}\ [1/(N-1)]^{1/2}\ \Sigma_i\ |\ I_{hkl,\ i} - <I_{hkl}>\ |\ /\ \Sigma_{hkl} <I_{hkl}>$

[2] $R_{work} = (\ \Sigma\ |\ |F_o| - |F_c|\ |\ )\ /\ (\ \Sigma\ |F_o|\ )$ - for all data except as indicated in footnote 3.

[3] 5% of data was used for the R_{free} calculation

Supplementary Table 3. CO3 TCR contacts with CD1a/β2m

TCR gene	TCR residues	CD1a residues	Bond type
CDR3γ	Tyr104	Trp23	VDW
FRγ	Arg48	His21	VDW
FRγ	Tyr51	His21, Ser22	VDW
FRγ	Leu61	His21	VDW
FRγ	Glu62	His21	VDW
FRγ	Ser63	His86	VDW
CDR3δ	Glu96-Oε1	Asn20-Nδ2	HB
CDR3δ	Glu96	Asn20	VDW
CDR3δ	Trp99-O	Asn20-Nδ2	HB
CDR3δ	Trp99	Tyr19	π-π stacking
CDR3δ	Trp99	Tyr19, Asn20, Trp23	VDW
CDR3δ	Pro100-O	Trp23-Nε1	HB
CDR3δ	Pro100	Trp23	VDW
CDR3δ	Asp101-Oδ1	Ser22-Oγ	HB
CDR3δ	Asp101	Asn20, His21, Ser22	VDW

TCR gene	TCR residues	β2m residues	Bond type
CDR1δ	Trp31	Asp35, Glu37, Asn84, His85, Val86	VDW
CDR3δ	Arg98-Nε	Asp35-Oδ2	HB
CDR3δ	Arg98-Nη2	Asp35-Oδ1	HB
CDR3δ	Arg98	Asp35, Glu37	VDW
CDR3δ	Trp99-N	Asp35-Oδ1	HB
CDR3δ	Trp99	Asp35	VDW

HB: Hydrogen bond, VDW: Van der Waals. Cut-off at 4 Å for VDW interactions and 3.5 Å for HB. FR, framework. Calculated using CCP4 contacts.

Supplementary Table 4. Exact values of the P values shown in Fig 7 c, d and e. The values were calculated using one-way ANOVA.

			**Exact P value**	P value stars
**Figure 7c**	TCR clusters per area	BK6-(ICAM-1) vs BK6-(ICAM-1+CD1a)		
		CO22-(ICAM-1) vs CO22-(ICAM-1+CD1a)	2.51E-18	****
		CO3-(ICAM-1) vs CO3-(ICAM-1+CD1a)	2.51E-18	****
**Figure 7c**	TCR cluster area	BK6-(ICAM-1) vs BK6-(ICAM-1+CD1a)	0.0949	ns
		CO22-(ICAM-1) vs CO22-(ICAM-1+CD1a)	4.03E-07	****
		CO3-(ICAM-1) vs CO3-(ICAM-1+CD1a)	0.9999	ns
**Figure 7c**	TCR localizations per cluster	BK6-(ICAM-1) vs BK6-(ICAM-1+CD1a)	0.1344	ns
		CO22-(ICAM-1) vs CO22-(ICAM-1+CD1a)	4.54E-05	****
		CO3-(ICAM-1) vs CO3-(ICAM-1+CD1a)	0.9998	ns
**Figure 7d**	pCD3zeta clusters per area	BK6-(ICAM-1) vs BK6-(ICAM-1+CD1a)	0.8302	ns
		CO22-(ICAM-1) vs CO22-(ICAM-1+CD1a)	1.46E-15	****
		CO3-(ICAM-1) vs CO3-(ICAM-1+CD1a)	0.9693	ns
**Figure 7d**	pCD3zeta cluster area	BK6-(ICAM-1) vs BK6-(ICAM-1+CD1a)	1.21E-18	****
		CO22-(ICAM-1) vs CO22-(ICAM-1+CD1a)	1.21E-18	****
		CO3-(ICAM-1) vs CO3-(ICAM-1+CD1a)	1.21E-18	****
**Figure 7e**	Proportion of TCR-pCD3z clusters above DoC threshold	BK6-(ICAM-1) vs BK6-(ICAM-1+CD1a)	3.57E-08	****
		CO22-(ICAM-1) vs CO22-(ICAM-1+CD1a)	3.57E-08	****
		CO3-(ICAM-1) vs CO3-(ICAM-1+CD1a)	3.57E-08	****
