# Supplementary material for: Atypical sideways recognition of CD1a by autoreactive γδ T cell receptors
Source: Nat Commun. 2022 Jul 5;13:3872. doi: 10.1038/s41467-022-31443-9 (PMC9256601; doi:10.1038/s41467-022-31443-9)
Supplement: Supplementary file 2 — Reporting Summary [file 41467_2022_31443_MOESM2_ESM.pdf]

nature portfolio

Corresponding author(s): Moody, van Rhijn & Rossjohn

Last updated by author(s): May 12, 2022

Reporting Summary

Nature Portfolio wishes to improve the reproducibility of the work that we publish. This form provides structure for consistency and transparency in reporting. For further information on Nature Portfolio policies, see our Editorial Policies and the Editorial Policy Checklist.

Statistics

For all statistical analyses, confirm that the following items are present in the figure legend, table legend, main text, or Methods section.

n/a	Confirmed	
☐	☒	The exact sample size (*n*) for each experimental group/condition, given as a discrete number and unit of measurement
☐	☒	A statement on whether measurements were taken from distinct samples or whether the same sample was measured repeatedly
☐	☒	The statistical test(s) used AND whether they are one- or two-sided<br>*Only common tests should be described solely by name; describe more complex techniques in the Methods section.*
☒	☐	A description of all covariates tested
☐	☒	A description of any assumptions or corrections, such as tests of normality and adjustment for multiple comparisons
☐	☒	A full description of the statistical parameters including central tendency (e.g. means) or other basic estimates (e.g. regression coefficient) AND variation (e.g. standard deviation) or associated estimates of uncertainty (e.g. confidence intervals)
☐	☒	For null hypothesis testing, the test statistic (e.g. F, t, r) with confidence intervals, effect sizes, degrees of freedom and P value noted<br>*Give P values as exact values whenever suitable.*
☒	☐	For Bayesian analysis, information on the choice of priors and Markov chain Monte Carlo settings
☒	☐	For hierarchical and complex designs, identification of the appropriate level for tests and full reporting of outcomes
☒	☐	Estimates of effect sizes (e.g. Cohen's *d*, Pearson's *r*), indicating how they were calculated

Our web collection on statistics for biologists contains articles on many of the points above.

Software and code

Policy information about availability of computer code

Data collection	The link to custom-build cluster analysis algorithm used in this study is available at GibHub repository link (https://github.com/PRNicovich/ClusDoC) which was originally published by Pageon et al., Mol Biol Cell 2016
Data analysis	All statistical analysis was done using GraphPad Prism software version 9.3.1. Crystallographic data were processed and analysed in XDS, CCP4 (4.71) and Phenix (1.19.2) software suites. SPR data were analysed using Scrubber 2. Flow cytometry data were analysed in FlowJo version 10.8.1.

For manuscripts utilizing custom algorithms or software that are central to the research but not yet described in published literature, software must be made available to editors and reviewers. We strongly encourage code deposition in a community repository (e.g. GitHub). See the Nature Portfolio guidelines for submitting code & software for further information.

Data

Policy information about availability of data

All manuscripts must include a data availability statement. This statement should provide the following information, where applicable:

- Accession codes, unique identifiers, or web links for publicly available datasets
- A description of any restrictions on data availability
- For clinical datasets or third party data, please ensure that the statement adheres to our policy

The structure factors and PDB coordinates of the crystal structures generated in this study have been deposited in the RCSB Protein Data Bank under the following accession codes: 7RYL https://doi.org/10.2210/pdb7RYL/pdb (CO3 binary), 7RYM https://doi.org/10.2210/pdb7RYM/pdb (CO3-CD1a-endo), 7RYN https://doi.org/10.2210/pdb7RYN/pdb (CO3-CD1a-sulfatide), 7RYO https://doi.org/10.2210/pdb7RYO/pdb (CO3-CD1a-DDM). The structural data used for molecular replacement in this study are available in the RCSB Protein Data Bank under accession codes 4X6B (https://doi.org/10.2210/pdb4X6B/pdb) and 7KP1 (https://

doi.org/10.2210/pdb7KP1/pdb). The surface plasmon resonance binding data generated in this study are provided in the Source Data file. T cell activation data and single cell image analyses are provided in the Source Data file. Source data are provided with this paper.

Field-specific reporting

Please select the one below that is the best fit for your research. If you are not sure, read the appropriate sections before making your selection.

- [x] Life sciences
- [ ] Behavioural & social sciences
- [ ] Ecological, evolutionary & environmental sciences

For a reference copy of the document with all sections, see nature.com/documents/nr-reporting-summary-flat.pdf

Life sciences study design

All studies must disclose on these points even when the disclosure is negative.

Sample size	In single-molecule imaging, single T cell sample size was kept at n≥30, sufficient for reporting statistical significance. For SPR binding all experiments were performed n≥2 times with 2 technical replicates each.
Data exclusions	No data points were excluded
Replication	All experiments were carried out at least twice. All attempts at replication were successful.
Randomization	Randomization was not relevant in the study because our experiments did not require allocation into groups.
Blinding	Quantifiable data was directly generated from the instruments, hence blinding investigator is not necessary

Reporting for specific materials, systems and methods

We require information from authors about some types of materials, experimental systems and methods used in many studies. Here, indicate whether each material, system or method listed is relevant to your study. If you are not sure if a list item applies to your research, read the appropriate section before selecting a response.

Materials & experimental systems

n/a	Involved in the study	
[ ]	[x]	Antibodies
[ ]	[x]	Eukaryotic cell lines
[x]	[ ]	Palaeontology and archaeology
[x]	[ ]	Animals and other organisms
[ ]	[x]	Human research participants
[x]	[ ]	Clinical data
[x]	[ ]	Dual use research of concern

Methods

n/a	Involved in the study	
[x]	[ ]	ChIP-seq
[ ]	[x]	Flow cytometry
[x]	[ ]	MRI-based neuroimaging

Antibodies

Antibodies used	CD3-brilliant violet (BV)421 (UCHT1; Biolegend), CD3-Fluorescein isothiocyanate (FITC) (SK7; BD Bioscience), CD3-allophycocyanin (APC; SP34-2, BD Biosciences), abTCR-phycoerythrin (PE) or FITC (clone T10B9, BD), gdTCR-PE (B1, Biolegend), CD69-BV650 (FN50, BioLegend). PE anti-human CD1a Antibody (Biolegend, HI149).
Validation	For the validation and titration of anti-CD3, -abTCR, -gdTCR, and -CD69 antibodies, PBMC were used. For the validation and titration of anti-CD1a, established stably transfected K562 cell lines were use as described in de Jong et al., Nat Imm 2010.

Eukaryotic cell lines

Policy information about cell lines

Cell line source(s)	HEK293T cells, parental K562 cells and the parental Jurkat cell lines were from ATCC. Transduced K562 and Jurkat cell lines were generated and validated in house.
Authentication	Jurkat76 cell lines were checked for TCR expression by staining with antiCD3 antibody<br>K562 cell line stably expressing CD1a was tested by staining with PE anti-human CD1a antibody
Mycoplasma contamination	Both parental cell lines, Jurkat76 and K562, tested negative for mycoplasma contamination before they were stably transduced. HEK293T are routinely tested for mycoplasma and were negative.

Commonly misidentified lines (See ICLAC register)	There were no commonly misidentified cell lines used in this study.

Human research participants

Policy information about studies involving human research participants

Population characteristics	We used discarded, de-identified leukoreduction collars provided by the Brigham and Women's Hospital Specimen Bank. We did not receive any data with the samples (including age, gender) so we do no know population characteristics.
Recruitment	These discarded samples were from random blood bank donors and not recruited specifically for this study.
Ethics oversight	These studies were approved by the Partners Healthcare Institutional Review Board.

Note that full information on the approval of the study protocol must also be provided in the manuscript.

Flow Cytometry

Plots

Confirm that:

- [x] The axis labels state the marker and fluorochrome used (e.g. CD4-FITC).
- [x] The axis scales are clearly visible. Include numbers along axes only for bottom left plot of group (a 'group' is an analysis of identical markers).
- [x] All plots are contour plots with outliers or pseudocolor plots.
- [x] A numerical value for number of cells or percentage (with statistics) is provided.

Methodology

Sample preparation	Jurkat76 and K562 cells were harvested and washed twice in phosphate buffered saline (PBS). Cells were then incubated with 1:500 diluted Zombie Aqua Fixable Viability Kit (cat. no. 423101, BioLegend, CA, USA) for 10 minutes (min) at room temperature (RT). Cells were washed in FACS buffer (i.e. PBS supplemented with 2% foetal calf serum (FCS) and 0.04 Sodium Azide (both Sigma-Aldrich, MO, USA) and stained with anti-human CD3-APC (clone SP34-2, cat. no. 557597) and CD69-BV650 (clone FN50, cat. no. 563835, both BD Biosciences, CA, USA) for 15 min on ice. After a wash in FACS buffer, they were fixed using IC Fixation Buffer (ThermoFisher Scientific) for 10 min at RT and washed.<br>Human peripheral blood mononuclear cells (PBMC), T cell lines and TCR-transfected 293T cells were stained with tetramers at 2 ug/ml in phosphate buffered saline (PBS) containing 1% bovine serum albumin (BSA) and 0.01% sodium azide. Cells and tetramer were incubated for 10 min at room temperature, followed by addition of unlabeled anti CD3 monoclonal antibody (OKT3) to a final concentration of 2 mg/ml for 10 min at room temperature, followed by addition of labelled antibodies and another incubation for 10 min at room temperature, followed by 10 min at 4 °C.
Instrument	Samples were acquired on a Fortessa X20 (BD Biosciences)
Software	Data was analysed in FlowJo (version 10.8.1; Treestar & BD Biosciences)
Cell population abundance	The number of sorted cells was too low for purity analysis directly post sorting. Cells were expanded as described before they were analysed after sorting. Sorting and post-sort analyses are shown in Fig. 1a, including the gate that was used to determine abundance.
Gating strategy	T cell activation assays: FSC-A/SSC-A > Singlets using FSC-A/FSC-H > Viable cells using FSC-A/Zombie Aqua > Cell Trace negative cells using FSC-A/Cell Trace > GFP positive cells using FSC-A/GFP > CD69/CD3<br>Tetramer-based sorting: FSC-A/SSC-A > Singlets using FSC-W/FSC-H > Singlets using SSC-W/SSC-H > gd T cells using CD3/abTCR

- [x] Tick this box to confirm that a figure exemplifying the gating strategy is provided in the Supplementary Information.
